# Insulin resistance uncoupled from dyslipidemia due to C-terminal PIK3R1 mutations

**DOI:** 10.1172/jci.insight.88766

**Published:** 2016-10-20

**Authors:** Isabel Huang-Doran, Patsy Tomlinson, Felicity Payne, Alexandra Gast, Alison Sleigh, William Bottomley, Julie Harris, Allan Daly, Nuno Rocha, Simon Rudge, Jonathan Clark, Albert Kwok, Stefano Romeo, Emma McCann, Barbara Müksch, Mehul Dattani, Stefano Zucchini, Michael Wakelam, Lazaros C. Foukas, David B. Savage, Rinki Murphy, Stephen O’Rahilly, Inês Barroso, Robert K. Semple

**Affiliations:** 1 The University of Cambridge Metabolic Research Laboratories, Wellcome Trust–MRC Institute of Metabolic Science, Cambridge, United Kingdom.; 2 The National Institute for Health Research Cambridge Biomedical Research Centre, Cambridge, United Kingdom.; 3 Metabolic Disease Group, Wellcome Trust Sanger Institute, Cambridge, United Kingdom.; 4 Wolfson Brain Imaging Centre, University of Cambridge, Cambridge, United Kingdom.; 5 National Institute for Health Research/Wellcome Trust Clinical Research Facility, Cambridge, United Kingdom.; 6 Inositide Laboratory, Babraham Institute, Cambridge, United Kingdom.; 7 Department of Molecular and Clinical Medicine, Sahlgrenska Academy at University of Gothenburg, Gothenburg, Sweden.; 8 Clinical Nutrition Unit, Department of Medical and Surgical Sciences, University Magna Graecia, Catanzaro, Italy.; 9 Department of Clinical Genetics, Glan Clwyd Hospital, Rhyl, United Kingdom.; 10 Department of Pediatrics, Children’s Hospital, Cologne, Germany.; 11 Section of Genetics and Epigenetics in Health and Disease, Genetics and Genomic Medicine Programme, UCL Institute of Child Health, London, United Kingdom.; 12 Pediatric Endocrine Unit, S.Orsola-Malpighi Hospital, Bologna, Italy.; 13 Institute of Healthy Ageing and Department of Genetics, Evolution and Environment, University College London, London, United Kingdom.; 14 Department of Medicine, Faculty of Medical and Health Sciences, University of Auckland, Auckland, New Zealand.

## Abstract

Obesity-related insulin resistance is associated with fatty liver, dyslipidemia, and low plasma adiponectin. Insulin resistance due to insulin receptor (INSR) dysfunction is associated with none of these, but when due to dysfunction of the downstream kinase AKT2 phenocopies obesity-related insulin resistance. We report 5 patients with SHORT syndrome and C-terminal mutations in *PIK3R1*, encoding the p85α/p55α/p50α subunits of PI3K, which act between INSR and AKT in insulin signaling. Four of 5 patients had extreme insulin resistance without dyslipidemia or hepatic steatosis. In 3 of these 4, plasma adiponectin was preserved, as in insulin receptor dysfunction. The fourth patient and her healthy mother had low plasma adiponectin associated with a potentially novel mutation, p.Asp231Ala, in adiponectin itself. Cells studied from one patient with the p.Tyr657X *PIK3R1* mutation expressed abundant truncated PIK3R1 products and showed severely reduced insulin-stimulated association of mutant but not WT p85α with IRS1, but normal downstream signaling. In 3T3-L1 preadipocytes, mutant p85α overexpression attenuated insulin-induced AKT phosphorylation and adipocyte differentiation. Thus, *PIK3R1* C-terminal mutations impair insulin signaling only in some cellular contexts and produce a subphenotype of insulin resistance resembling INSR dysfunction but unlike AKT2 dysfunction, implicating PI3K in the pathogenesis of key components of the metabolic syndrome.

## Abstract 2

C-terminal mutations in human PIK3R1 are associated with severe insulin resistance in the absence of dyslipidemia or hepatic steatosis.

## Introduction

Insulin exerts effects on cells via a widely ramifying signaling network, critically involving phosphorylation of insulin receptor substrate (IRS) proteins by activated insulin receptor, recruitment of class 1A PI3K to the phosphorylated IRS proteins, and thus activation of the AKT serine threonine kinases by 2 phosphoinositide-dependent kinases (PDK1 and -2) (1). This signaling cascade culminates in stimulation of glucose uptake by skeletal muscle and adipose tissue, suppression of hepatic gluconeogenesis, and stimulation of hepatic lipogenesis among other responses.

Attenuation of insulin’s glucose-lowering action in vivo, termed “insulin resistance,” commonly develops as a maladaptive response to obesity and is widely viewed as a critical mediator of the link between obesity and pandemic disorders, including type 2 diabetes, fatty liver, dyslipidemia, subfertility, and cancer. However, focusing exclusively on insulin resistance while neglecting to consider whether the manifold actions of insulin on other aspects of metabolism and cell growth are commensurately impaired is potentially misleading. The tight negative feedback loop coupling blood glucose concentration to insulin secretion means that the glucose-lowering action of insulin determines blood insulin concentration. Defects limited to this glucose-lowering action thus produce compensatory hyperinsulinemia, exposing any less-resistant insulin-responsive pathways or tissues to high levels of insulin action.

The concept that such “partial” insulin resistance is involved in the pathogenesis of insulin resistance–related disease has been discussed since at least the 1980s, when it was invoked to account theoretically for the association between hypertension and insulin resistance (2). Direct experimental evidence for the phenomenon was provided in type 2 diabetes and obesity in ensuing years (3–6). Further murine studies have since produced data consistent with this general concept (7), which is particularly well developed in the case of insulin action on endothelial cell function (8).

More recently, studies of humans with single gene disorders of proximal insulin signaling have supported the importance of partial insulin resistance: genetic defects in the insulin receptor, although replicating in severe form many features of common insulin resistance (hyperglycemia, extreme hyperinsulinemia, ovulatory dysfunction, hyperandrogenism, acanthosis nigricans, and soft tissue overgrowth), do not feature other critical components of the syndrome (elevated plasma triglycerides, suppressed HDL cholesterol, fatty liver [refs. 9, 10], and suppressed plasma adiponectin [ref. 11]). In contrast, in a single family with a loss-of-function mutation in the serine threonine kinase *AKT2*, a critical more distal component in insulin signaling, affected patients had a severe phenocopy of the prevalent metabolic syndrome, encompassing severe fatty liver, dyslipidemia, and suppressed plasma adiponectin (9). It is not clear at present whether the preserved or increased plasma adiponectin seen in insulin receptor dysfunction may play a role in the protection from fatty liver and dyslipidemia in that state. The metabolic consequences in humans of primary genetic defects in class 1A PI3K, which lies between the insulin receptor and AKT2 in the insulin signaling pathway, have not been evaluated in similar detail.

Class 1A PI3K is a heterodimer of one of 3 p110 catalytic subunits — p110α, p110β and p110δ — and one of 5 regulatory subunits, three of which, namely p85α, p55α, and p50α, are splice variants derived from the *PIK3R1* gene (12). Regulatory subunits harbor 2 phosphotyrosine-binding SH2 domains and thus play a critical role in PI3K recruitment to phosphorylated receptor tyrosine kinases (RTKs) or IRS proteins. Pharmacological studies have implicated p110α most strongly in insulin action in adipocytes, with p110β suggested to play a lesser, accessory role (13). In keeping with this, although total knockout of either p110α (14) or p110β (15) is embryonically lethal, heterozygous knockin of a kinase-dead p110α allele (16), or liver-specific ablation of p110β (17), produces insulin resistance in early life. In contrast, knockout of either p85α (18) or p85β (19) produces a phenotype of increased insulin sensitivity, while knockout of p85α, p55α, and p50α together is lethal (20). Collectively these studies argue for a critical role for PI3K generally in insulin action, but suggest that the role of the regulatory subunits in particular is more complex.

Activating mutations in *PIK3R1* have been described in cancers and, more recently, in an immunodeficiency syndrome featuring severely reduced plasma immunoglobulin levels (21, 22). A single patient with selective genetic loss of the p85α isoform has also been described with agammaglobulinemia (23); however, no systemic metabolic defects have been reported in these disorders. In 2013, mutations in *PIK3R1* were also demonstrated to cause SHORT syndrome, denoting short stature, joint hyperextensibility, ocular depression, Rieger anomaly (a developmental defect in the iris), and teething delay (24–26). Lipodystrophy is also reported to be common in SHORT syndrome, while insulin resistance has been reported in many, though not all, cases (27). Limited studies of insulin action in patient-derived cells has yielded inconsistent findings, though these were interpreted as indicative of cellular insulin resistance (24–26).

In this report we describe the more detailed metabolic and cellular phenotype of 5 patients with pathogenic C-terminal *PIK3R1* mutations, one of whom had an additional rare missense mutation, p.Asp231Ala, in the *ADIPOQ* gene and severely suppressed plasma adiponectin concentration.

## Results

### A syndrome of normolipidemic insulin resistance without immunodeficiency.

**Figure 1:**
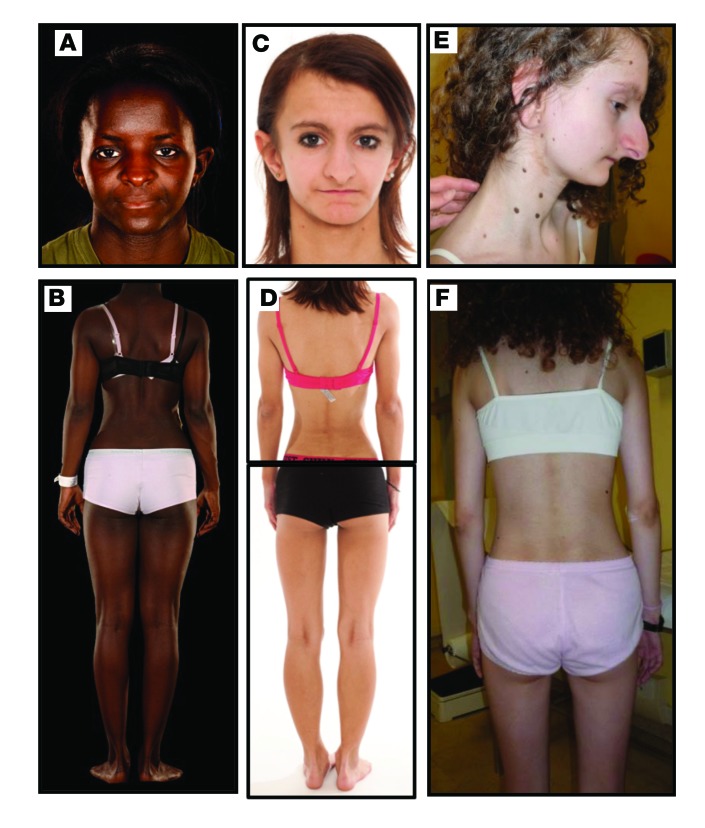
Appearance of patients with severe insulin resistance and mutations in *PIK3R1*. (**A**) Facial appearance of Patient 1 (P1) at 16 years old. (**B**) Posterior view of P1 showing absence of lipodystrophy. (**C**) Facial appearance of P2 aged 13 years old. (**D**) Composite posterior view of P2 showing lean build with generalized paucity of adipose tissue. (**E**) Facial view of P3 at 13 years old. (**F**) Posterior view of P3 showing lean build with generalized paucity of adipose tissue.

The female proband (patient 1 [P1]) was born to non-consanguineous parents of Nigerian origin with a birth weight of 2.54 kg at term (–2.4 standard deviation scores [SDS]). At 12 years old she presented with symptomatic hyperglycemia during a febrile illness. Biochemical evaluation on recovery revealed extreme fasting hyperinsulinemia of 1,140 pmol/l but normal fasting glucose. On assessment at 16.2 years, she reported coarse facial hair growth and primary amenorrhea but no other symptoms. Mild facial dysmorphism was noted, including deep-set eyes, a triangular face with small jaw, and low-set ears (Figure 1A). She was of short stature compared with her parents (height 1.56 m; mid-parental height 1.77 m) and lean (BMI 18.6 kg/m^2^, SDS –0.821) but not lipodystrophic (Figure 1B), with marked flexural acanthosis nigricans, well advanced puberty, and mild facial hirsutism. Fasting blood glucose was normal, but diabetes was found after oral glucose challenge, with extreme hyperinsulinemia. Fasting serum triglycerides, LDL and HDL cholesterol, and leptin were normal, while plasma adiponectin was extremely low (Table 1). Testosterone was severely elevated, and pelvic ultrasound revealed polycystic ovaries. Biochemical indices of liver function and hepatic ultrasound were normal. Treatment with metformin and a combined cyproterone acetate/ethinylestradiol preparation was begun.

Dual-energy X-ray absorptiometry (DXA) revealed a total body fat content of 24.8%, with normal distribution. Proton magnetic resonance spectroscopy (MRS) detected an intrahepatic triglyceride content of 0.1%, well below the sex- and ethnicity-matched interquartile range (2.7%–8.9%) for insulin-resistant patients from a large, population-based study (28). Intramuscular lipid content was normal. Fasting de novo lipogenesis in the liver, assessed by incorporation of deuterium into VLDL, was virtually undetectable at 0.004%. As severe insulin resistance without dyslipidemia or fatty liver is characteristic of human insulin receptoropathy, the *INSR* gene was sequenced and found to be normal, and there was no evidence of anti-insulin receptor antibodies on IP assay.

Both parents were of normal stature, obese, but without severe insulin resistance. The mother of the proband (M1) also had very low plasma adiponectin concentrations, while the father (F1) had impaired fasting glucose and impaired glucose tolerance on glucose challenge (Table 1).

### Genetic evaluation of proband.

We hypothesized that the syndrome was caused by either biallelic loss-of-function mutations (most likely compound heterozygous), or a de novo dominant mutation, in a critical insulin signaling–related gene. To address this, we performed exome-wide sequencing of the proband (P1) and her parents and undertook complementary analyses: a search for de novo mutations in the proband using DeNovoGear (29) and a search for genes with putative compound heterozygous rare variants. Resulting variant lists were filtered to exclude variants unlikely to alter protein function or those common in control populations. Three de novo variants but no compound heterozygous variants remained after filtering and validation by Sanger sequencing in the family trio (Supplemental Table 1; supplemental material available online with this article; doi:10.1172/jci.insight.88766DS1).

**Figure 2:**
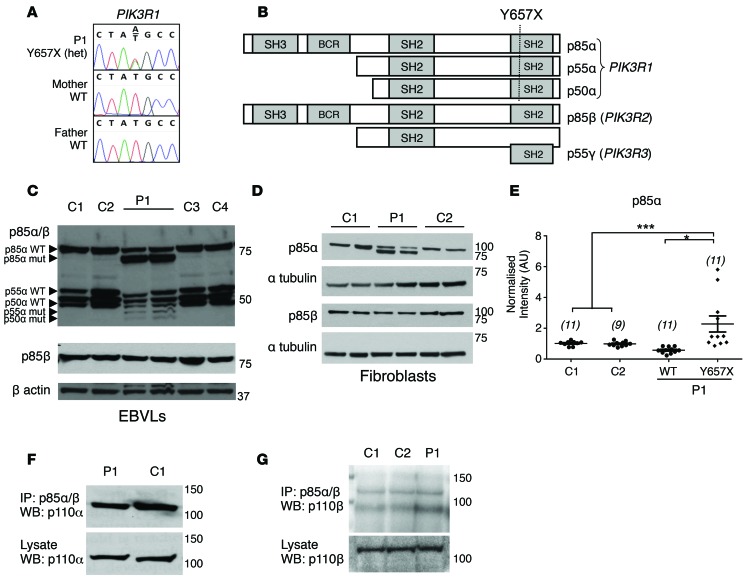
A de novo heterozygous nonsense mutation in *PIK3R1* in a patient with severe insulin resistance*.* (**A**) Genomic DNA sequence from patient 1 (P1) and her parents showing the de novo p.Tyr657X (Y657X) mutation in *PIK3R1*. (**B**) Domain structure of the protein products of *PIK3R1* (p85α, p55α, and p50α) and predicted truncation site resulting from the nonsense mutation. (**C**) Full-length and truncated p85α, p55α, and p50α (indicated by arrowheads) and full-length p85β in EBV-transformed lymphoblastoid cells (EBVLs) from P1 and healthy controls (C1–C4), as assessed by Western blotting for total p85 (p85α/β) or p85β alone. (**D**) Expression of p85α or p85β in dermal fibroblasts from P1 and healthy controls (C1, C2). (**E**) Quantification of WT and mutant p85α expression in P1 and control fibroblasts from 4 independent Western blots, normalized to mean expression of WT p85α in controls. *P < 0.05, ***P < 0.005; 1-way ANOVA followed by post-hoc Tukey test. Total number of observations indicated in parentheses. Dot plot displays mean ± SEM. (**F**) Western blot of p110α after IP of total p85 in primary dermal fibroblast lysates. (**G**) Western blot of p110β after IP of total p85 in primary dermal fibroblast lysates.

Of the 3 de novo variants, a heterozygous nonsense mutation in *PIK3R1*, encoding the p85α, p55α, and p50α regulatory subunits of PI3K, stood out. The mutation, p.Tyr657X (Y657X) when numbered according to the p85α gene product, was predicted to remove the C-terminal 68 amino acids from all 3 gene products, corresponding to two-thirds of the C-terminal SH2 domain (Figure 2, A and B). No further variants in *PIK3R1* were identified, ruling out compound heterozygosity.

### Expression and heterodimerization of mutant PIK3R1.

Sequencing of cDNA from EBV-transformed lymphoblastoid cells derived from the proband revealed approximately equal expression of WT and mutant mRNA, with no difference in total *PIK3R1* transcript levels, indicating normal expression and stability of the mutant mRNA. There was no evidence of compensatory upregulation of *PIK3R2* or *PIK3R3* mRNA, encoding the p85β and p55γ regulatory subunits, respectively (Supplemental Figure 1). Immunoblotting using an antibody with affinity for all regulatory subunit isoforms showed abundant expression of truncated p85α protein in addition to full-length protein, while truncated p55α and p50α were also detectable (Figure 2C). Immunoblotting of lysates from dermal fibroblasts with a p85α-specific antibody showed both full-length and truncated p85α, while p85β was present only in the WT form, with no evidence of compensatory upregulation (Figure 2, D and E). In fibroblasts from the proband, the truncated p85α isoform was consistently more abundant than its WT counterpart, assessed using both the total p85 antibody and the p85α-specific antibody. Moreover, WT p85α was expressed at lower levels in proband cells compared with controls (Figure 2, D and E). There was no apparent defect in the capacity of p110α to dimerize with p85, as assessed by IP of p85 and immunoblotting for p110α in either primary dermal fibroblasts (Figure 2F) or HEK293-T cells transiently overexpressing HA-tagged p85α and myc-tagged p110α (Supplemental Figure 2A). Dimerization with p110β in proband fibroblasts also appeared normal (Figure 2G).

### Identification and metabolic phenotyping of further affected individuals.

*PIK3R1*, *PIK3R2*, and *PIK3CA*, encoding the p110α catalytic subunit, were next sequenced in 262 further probands with severe insulin resistance. This revealed 3 individuals (P2–P4) all with the same rare missense variant, p.Arg649Trp (R649W), in the C-terminal SH2 domain of *PIK3R1*. One further patient (P5) was later identified based on syndromic features and confirmed also to have the same mutation. (For additional information regarding P2–P5, see Supplemental Case Studies.) In all cases, the rare *PIK3R1* variant was shown to be de novo on sequencing parental DNA. Other rare sequence variants were also discovered in each of the genes studied; however, none of these were both predicted to be deleterious and cosegregated with severe insulin resistance in the families studied, so were not studied further (Supplemental Table 2).

While this study was underway, 3 groups independently reported C-terminal *PIK3R1* mutations, including both p.Tyr657X and p.Arg649Trp, to be the cause of SHORT syndrome (24–26). All 5 patients with *PIK3R1* mutations we describe had facial features in keeping with SHORT syndrome, as well as varying numbers of other previously described features (Supplemental Table 3). Although P1 had no lipodystrophy, P2, P3, and P4 had general paucity of subcutaneous fat, which was more apparent postpubertally (P2 and P3 are shown in Figure 1). Although P5 also had sparse subcutaneous adipose tissue, it is difficult to discriminate this from healthy leanness in a prepubertal boy. All patients had leptin levels toward the lower end of the normal range (Table 1), consistent with low adipose mass. In all cases, immunoglobulin levels and blood cell counts were normal, and no patient had a clinical history suggestive of immunodeficiency (Supplemental Table 3).

We next assessed whether the 4 additional patients with *PIK3R1* mutations shared the insulin resistance subphenotype of the proband. Three of the 4 additional patients (P2–P4), females between 12 and 14 years old at the time of evaluation, had severe insulin resistance, yet, like P1, they showed no dyslipidemia or evidence of fatty liver (Table 1). Unlike P1, however, they each had strikingly preserved plasma adiponectin levels, with values above the mean for females. This finding, in the context of severe insulin resistance, was previously considered to be pathognomonic of insulin receptor loss of function (11, 30, 31). The final patient (P5), a prepubertal boy of 9 years old, was evaluated in less detail in the nonfasting state and showed neither clinical nor biochemical evidence of severe insulin resistance or dyslipidemia.

### Cellular consequences of PIK3R1 truncation.

**Figure 3:**
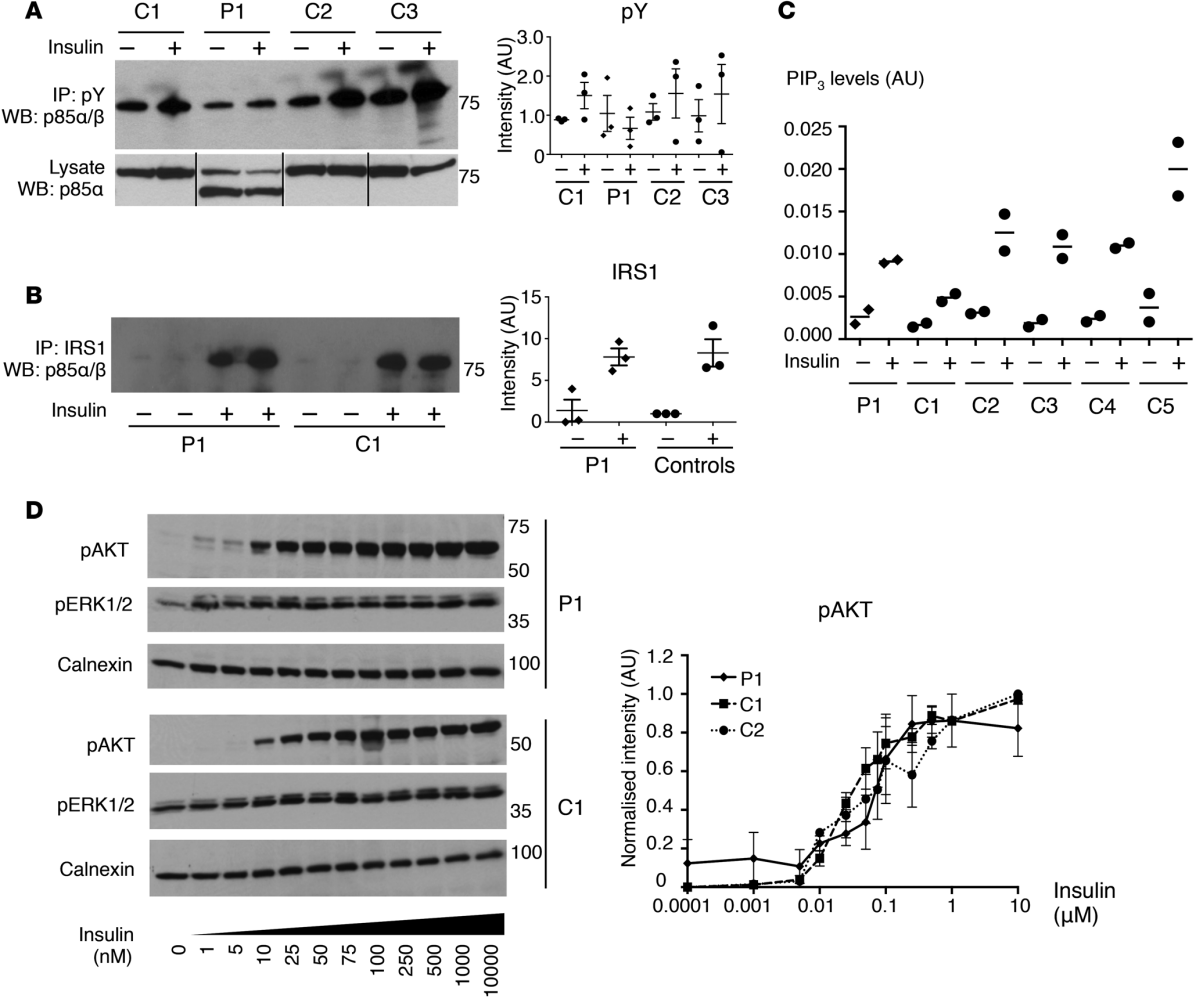
Downstream insulin signaling in patient cells expressing truncated p85α. (**A**) Western blot of total p85 (p85α/β) in phosphotyrosine immunoprecipitates or total lysates from dermal fibroblasts from P1 and healthy controls (C1–C3) treated with PBS or 100 nM insulin. Representative blots with quantified data from 3 independent experiments normalized to mean baseline intensity (dot plot displays mean ± SEM). Lane order in image of lysate blot adjusted to reflect that of the immunoprecipitate blot. (**B**) Western blot of total p85 (p85α/β) in phospho-IRS1 immunoprecipitates from patient and control fibroblasts after treatment with PBS or insulin. Representative blot with quantified data from three independent experiments normalized to mean baseline intensity across control cell lines. (**C**) Phosphatidylinositol-3,4,5-trisphosphate (PIP_3_) levels in dermal fibroblasts from P1 and healthy controls (C1–C5) stimulated with PBS or insulin, as determined by liquid chromatography mass spectroscopy. PIP_3_ area ratios were normalized to that of an internal standard. Dot plot displays median of 2 samples, each quantified in duplicate. Representative of 3 independent experiments. (**D**) Western blot of phosphorylated AKT1/2 (Ser473/474) and ERK1/2 (Thr202/Tyr204) in dermal fibroblasts from P1 and healthy controls (C1, C2) after stimulation with a range of insulin doses. Representative blots with quantified data from 3 independent experiments normalized to the highest intensity signal in each experiment and a calnexin loading control. Data represent mean ± SEM.

Although the N-terminal SH2 domain in *PIK3R1* gene products is believed to play the dominant role in phosphotyrosine binding, some studies have suggested that the C-terminal domain also plays a significant role (32). In human primary dermal fibroblasts from healthy controls, p85 was abundant in phosphotyrosine immunoprecipitates, and this showed a trend toward an increase after insulin stimulation (Figure 3A). In contrast, no mutant p85 was detectable in phosphotyrosine immunoprecipitates from fibroblasts from P1 either in the basal or insulin-stimulated state, in spite of a high level of expression in the corresponding lysates. No increase in full-length p85 appearance in phosphotyrosine complexes was seen after insulin stimulation in P1 (Figure 3A). Insulin stimulation did lead to the appearance of p85 in IRS1 immunoprecipitates in control cells, but no mutant p85 could be detected in immunoprecipitates from cells from P1. Recruitment of full-length p85 to IRS1 in response to insulin in the same cells was not reduced, however (Figure 3B). Finally, in HEK293-T cells transiently overexpressing HA-tagged WT or mutant p85α, together with myc-tagged p110α, less HA-tagged mutant p85 coimmunoprecipitated with phosphotyrosine than WT HA-p85, and insulin-induced association of mutant but not WT p85 was abolished (Supplemental Figure 2B).

These findings in primary cells and a cell line heterologously expressing p85 suggest that recruitment of the mutant p85 to tyrosine-phosphorylated IRS1 is severely suppressed or absent; however, in primary skin cells from the proband, this does not impair recruitment of coexpressed WT p85. Insulin-induced generation of phosphatidylinositol-3,4,5-trisphosphate (PIP_3_) and phosphorylation of the key downstream substrate AKT, a PIP_3_-dependent process, were next assessed in patient cells. The insulin-induced increase in PIP_3_ levels (Figure 3C) and AKT phosphorylation (Figure 3D) showed no difference between patient and control dermal fibroblasts, even when the latter was examined in response to a wide range of different insulin doses. There was, however, a nonsignificant increase in basal AKT phosphorylation (Figure 3D). Furthermore there was no evidence of a faster rate of AKT dephosphorylation after ligand was washed off the cells (Supplemental Figure 3), and both WT and mutant p85α coimmunoprecipitated with the PIP_3_ phosphatase PTEN (Supplemental Figure 4).

**Figure 4:**
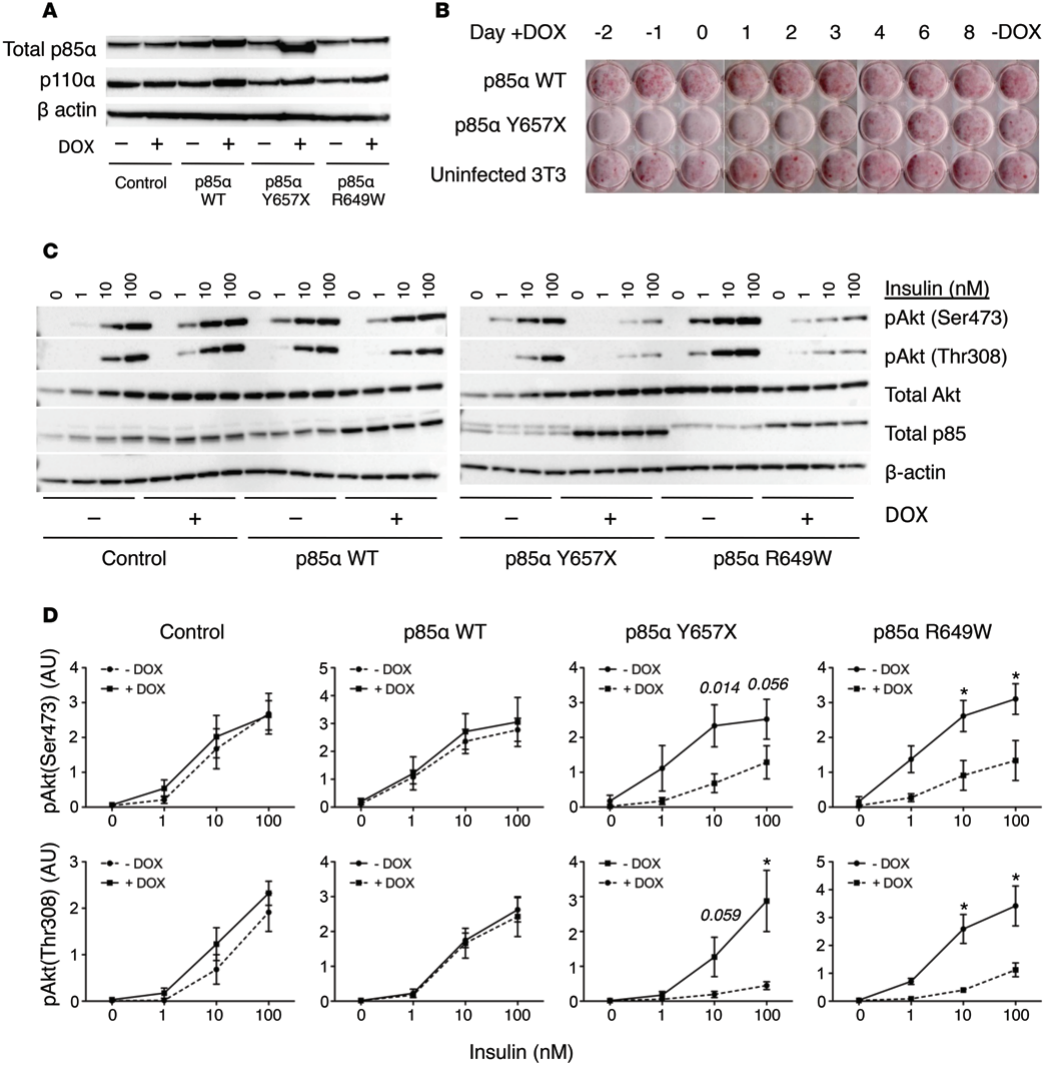
Insulin signaling and adipocyte differentiation in 3T3-L1 preadipocyte models of SHORT syndrome. (**A**) Total p85 and p110α expression in preadipocyte lysates 72 hours after treatment with or without doxycycline (DOX) to induce expression of WT or mutant p85α. β Actin was assessed as a loading control. (**B**) Lipid accumulation, assessed by Oil red O staining on day 10 of an adipocyte differentiation protocol. Doxycycline was added at the time points indicated to inducibly overexpress full-length or truncated p85α. (**C**) Insulin-induced phosphorylation of AKT at Ser473/474 and Thr308 in preadipocytes infected with WT and mutant p85α lentivirus, with or without doxycycline treatment, after stimulation with 1, 10, or 100 nM insulin for 10 minutes. Representative of 3 independent experiments. (**D**) Quantification of AKT phosphorylation, showing pooled data from 3 independent experiments. –DOX and +DOX groups were compared using multiple *t* tests, with correction for multiple comparisons using the Holm-Šidák method, with α = 0.05. Data represent mean ± SEM; *P < 0.05.

In order to assess the effect of the p85α truncation mutation in a canonically insulin-responsive cell type, lentiviral constructs were used to generate 3T3-L1 murine preadipocytes showing doxycycline-inducible overexpression of WT p85α or one of two p85α mutants — the p.Tyr657X mutation studied in primary cells and the p.Arg649Trp mutation, which accounts for around 50% of SHORT syndrome (Figure 4A). Overexpression of mutant but not WT p85α in preadipocytes impaired differentiation of cells to adipocytes on exposure to a standard adipogenic regimen (data shown for Y657X in Figure 4B) and markedly attenuated insulin-induced AKT phosphorylation (Figure 4, C and D).

### A dominant negative mutation in ADIPOQ.

**Figure 5:**
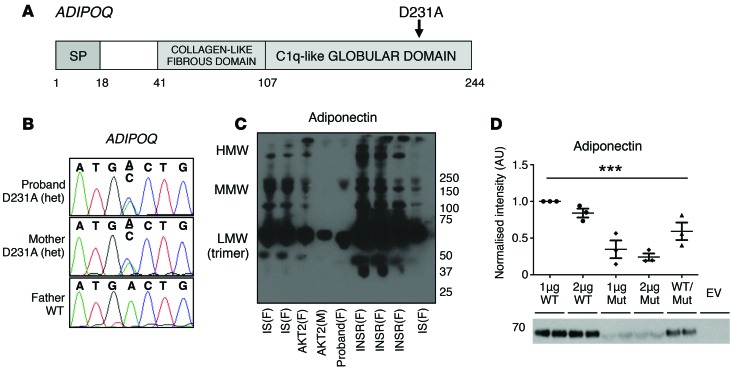
A dominant negative mutation in *ADIPOQ.* (**A**) Domain structure of adiponectin, with the approximate site of the p.Asp231Ala (D231A) substitution indicated. SP, signal peptide. (**B**) Genomic DNA sequence from P1 and her parents showing the D231A mutation in *ADIPOQ*. (**C**) Western blot of adiponectin in serum from P1, insulin-resistant controls with mutations in *INSR* or *AKT2*, and insulin-sensitive controls (IS), after non-denaturing, non-reducing SDS-PAGE. Representative of 3 independent experiments. Low- (LMW), medium- (MMW), and high-molecular-weight (HMW) adiponectin complexes are indicated. F, female; M, male. (**D**) Secretion of low-molecular-weight adiponectin complexes into the culture medium of HEK293-T cells transfected with 1 μg or 2 μg WT or mutant *ADIPOQ* or cotransfected with 1 μg WT and 1 μg mutant *ADIPOQ*, determined by non-denaturing, non-reducing SDS-PAGE, followed by Western blotting of adiponectin. Representative blot with quantified data from 3 independent experiments (*n* = 2 in each experiment), normalized to intensity of the 1 μg WT sample. Dot plot displays mean ± SEM. ***P < 0.005; 1-way ANOVA. EV, empty vector.

In view of the discordance in plasma adiponectin values between P1 and the 3 other patients with *PIK3R1* mutations and extreme insulin resistance, we reexamined the exome sequence of P1 to determine whether there were any sequence variants in the *ADIPOQ* gene, encoding adiponectin. Both she and her mother, who also had severely suppressed serum adiponectin, were found to harbor what we believe to be a novel heterozygous missense mutation, p.Asp231Ala, within the globular domain of adiponectin; this mutation was predicted to be deleterious by multiple algorithms, and was absent from 11,532 control exomes and genomes as well as from the 60,706 individuals in the Exome Aggregation Consortium (ExAC) database (33) (Figure 5, A and B).

Non-denaturing, non-reducing Western blotting of serum from P1 revealed that expression of all adiponectin species was markedly lower compared with healthy controls and patients with genetic defects in the insulin receptor (Figure 5C). Instead, adiponectin levels were comparable to that of a patient with a loss-of-function mutation of *AKT2*. HEK293-T cells transiently transfected with mutant human *ADIPOQ* expressed and secreted lower levels of adiponectin compared with those overexpressing the WT protein, as assessed by Western blotting of cellular lysates or medium (Figure 5D). Moreover, cotransfection of WT and mutant adiponectin resulted in lower levels of adiponectin secretion compared with transfection with WT adiponectin alone, indicating that the mutant *ADIPOQ* exerts a dominant negative effect over its WT counterpart (Figure 5D). Collectively, these data suggest that the *ADIPOQ* mutation causes primary hypoadiponectinemia in P1 and her mother. Importantly, although the proband had extreme insulin resistance, her mother had a benign metabolic profile despite being obese (Table 1).

## Discussion

The clinical syndromes and *PIK3R1* mutations we describe are consistent with previous reports of SHORT syndrome (24–26, 34). Four of the 5 patients we describe exhibited extreme insulin resistance, while the fifth patient was prepubertal and male, which may explain his much milder metabolic phenotype. It has previously been shown that patients with *INSR* mutations, despite severe insulin resistance, do not show fatty liver, dyslipidemia, or suppressed plasma adiponectin, while patients from the single family described to date with severe insulin resistance ascribed to an *AKT2* mutation exhibit all these to a severe degree (9). The insulin resistance subphenotype of SHORT syndrome patients, who have a functional defect lying between INSR and AKT2 in the insulin signaling pathway, closely resembles that seen in INSR dysfunction. The only exception in this report was P1, who showed severely suppressed plasma adiponectin, which we suggest is attributable to the *ADIPOQ* p.Asp231Ala mutant identified here. Our findings suggest that normolipidemic severe insulin resistance without fatty liver and with preserved or elevated adiponectin may be indicative of proximal insulin signaling defects generally, rather than loss of receptor function specifically.

One possible explanation for the discordance between *INSR*- and *AKT2*-associated human insulin resistance subphenotypes lies in the pronounced femorogluteal lipodystrophy associated with *AKT2* mutation, albeit only in a single family to date (9), but not with *INSR* mutations. This raises the possibility that AKT2 is more important for insulin action in adipocytes than in hepatocytes, so that partial loss of its function is expressed as a predominantly “lipodystrophy-like” metabolic phenotype. However SHORT syndrome, too, has been reported to be associated with a pattern of lipodystrophy including loss of femorogluteal adipose depots (24–26). This was not seen in P1 in this study; however, each of the other severely insulin-resistant patients we describe showed generalized paucity of subcutaneous fat. As further cases are studied, it will be important to assess whether SHORT syndrome does uncouple femorogluteal lipodystrophy from dyslipidemia and fatty liver, uniquely among lipodystrophy syndromes affecting femorogluteal depots.

Class 1A PI3K has convincingly been shown to mediate insulin’s metabolic actions; however, study of the specific roles of regulatory subunits has been challenging. In contrast to the severe insulin resistance seen in SHORT syndrome, haploinsufficiency or complete knockout of p85α alone (18), p85α, p55α, and p50α together (20, 35), p55α and p50α together (36), or p85β (19) all increase insulin sensitivity in mice. Liver-specific p85α knockout also increased insulin sensitivity (37), while p85α haploinsufficiency protected mice with concomitant heterozygous knockout of the insulin receptor and Irs1 from overt diabetes (35). Only when p85α and p85β were knocked out together in the liver were insulin resistance, glucose intolerance, and hypolipidemia — as in the patients we describe — seen (38). One model advanced to explain this suggests that regulatory subunits are expressed in excess of catalytic subunits, producing free regulatory subunits that can compete for phosphotyrosine binding with catalytically competent heterodimers, and also sequester IRS1. Reducing expression of regulatory subunits selectively reduces the free subunits, and thus tightens the coupling of insulin stimulation to PI3K activation (39). However, several lines of evidence against this model have been advanced, leading to the alternative hypothesis that reduced activity of negative regulators of the PI3K pathway such as PTEN accounts for the increased insulin sensitivity that results from regulatory subunit deletion (40).

Previous signaling studies of human SHORT-related *PIK3R1* mutations in primary cells have been inconsistent and limited by use of single WT control lines (24–26) and/or by use of lymphoblastoid cells transformed by EBV (26), which is known to activate PI3K (41). One study reported only a baseline increase in downstream signaling (25), while another showed no basal difference but a very modest decrease in peak stimulation of IRS1 and phosphotyrosine-associated PI3K activity (24). In Pik3r1-null murine fibroblasts in which WT or mutant (p.Arg649Trp) PIK3R1 was stably overexpressed, a striking reduction in IRS1-associated PI3K activity was seen in response to insulin, while, in contrast, basal phosphotyrosine-associated PI3K was increased, with no difference in stimulated activity (24). Our more detailed study in primary dermal fibroblasts using multiple control lines suggests that PI3K signaling in response to insulin is not impaired, but replicates the observation that association of the mutant p85α with IRS1 in response to insulin is severely reduced.

Our findings afford the opportunity to test in humans the “free regulatory subunit” hypothesis advanced to explain enhanced insulin sensitivity in heterozygous p85α-knockout and related mice. Truncated p85α was increased in the primary cells of P1, likely due to loss of a recently described C-terminal ubiquitylation motif (42). However, previous studies have shown that the p.Arg649Trp variant found in the other patients we describe does not show increased expression in primary cells, suggesting that increased regulatory subunit levels are not a generalized correlate of insulin resistance in SHORT syndrome. Critically, we also show that the ability of the truncated p85α to be recruited to IRS1 in response to insulin is severely impaired or absent in primary fibroblasts, consistent with prior studies in Pik3r1-null murine fibroblasts stably overexpressing the p.Arg649Trp mutant PIK3R1 (24); however, recruitment of full-length WT p85α to IRS1 is normal, as are insulin-stimulated PIP_3_ generation and AKT phosphorylation in that cell type. It thus seems unlikely that competition between mutant and WT p85α explains the insulin resistance of SHORT syndrome. It remains possible, however, that in key insulin-responsive tissues in humans, reduction of recruitable heterodimeric class 1A PI3K does impair insulin signaling, even though no effect could be discerned in dermal fibroblasts. In keeping with this, overexpression of truncated but not WT p85α in 3T3-L1 murine preadipocytes did attenuate insulin-induced AKT phosphorylation and impair differentiation to adipocytes; however, this will require verification in insulin-responsive cells with endogenous levels of mutant expression given the potential confounding effect of perturbed stoichiometry in overexpression systems. Indeed, while this article was being prepared, a knockin mouse heterozygous for the *Pik3r1* p.Arg649Trp mutation was reported. While this exhibited insulin resistance in vivo and impaired proximal insulin signaling in vitro, no impairment of preadipocyte differentiation ex vivo was reported (43).

Our finding in P1 and her mother of a missense mutation in the *ADIPOQ* gene that impairs adiponectin secretion and acts in *trans* in a cell culture model to impair secretion of coexpressed WT *ADIPOQ* is significant in two respects. First, the fact that low liver fat, normal lipid profile, and nearly undetectable hepatic de novo lipogenesis were still seen in P1 despite concomitant genetic suppression of adiponectin argues that the apparent protection from these components of the metabolic syndrome in SHORT syndrome — and probably by extension also in insulin receptor defects — is not accounted for by the preserved or elevated plasma adiponectin seen in these forms of severe insulin resistance. Second, the normal insulin sensitivity of the mother of the proband, despite obesity and genetic suppression of plasma adiponectin levels, suggests that primary adiponectin deficiency does not lead to highly penetrant insulin resistance. This adds to the debate about the metabolic importance of adiponectin in humans. Evidence from murine models suggests that adiponectin exerts insulin-sensitizing effects; however, human evidence is mixed and open to different interpretations (44). Indeed, the largest Mendelian randomization study reported failed to provide evidence for a causal role of low plasma adiponectin in insulin resistance (45). In contrast, two smaller Mendelian randomization studies supported a causal link (46, 47), while a recently described family with a different, novel missense mutation that conferred a lesser degree of adiponectin suppression than we describe, did show cosegregation between suppressed adiponectin and insulin resistance (48). As increasing numbers of rare *ADIPOQ* alleles are identified in large-scale exome sequencing studies, it will be of value to identify any that suppress plasma adiponectin and to study metabolic traits in the carriers.

None of the patients we describe had clinical or laboratory evidence of immunodeficiency or hypogammaglobulinemia. These have been associated both with germline activating mutations in *PIK3R1* (21, 22) and, in a single patient, with a germline homozygous mutation abolishing p85α expression, with preservation of p55α and p50α (23). The lack of immunodeficiency in SHORT syndrome argues that in B cell development, p85α plays an essential role that cannot be complemented by p55α and p50α, and that moreover does not depend critically on the C-terminal SH2 domain, which we show in other cell types is critical for p85 recruitment to IRS1 and phosphotyrosine-containing complexes.

In summary, our findings establish that in humans, C-terminal SH2 domain mutations in *PIK3R1* produce a metabolic phenocopy of insulin receptor dysfunction, with strikingly preserved liver fat, lipid profile, and plasma adiponectin despite severe insulin resistance. Unlike deficiency of p85α, however, they do not produce immunodeficiency. We demonstrate that one SHORT-associated mutation studied shows severely reduced recruitment to IRS1 in response to insulin in skin fibroblasts, but that this does not impair downstream PIP_3_ generation or AKT phosphorylation in the same cells. In contrast, overexpression of the same mutant in murine preadipocytes, as in a recently described knockin mouse model, does attenuate AKT phosphorylation in response to insulin stimulation. This demonstrates that the effect of this SHORT syndrome *PIK3R1* mutation on insulin signaling is cell type specific. Further studies will be required to understand fully how SHORT syndrome–associated *PIK3R1* mutations produce severe insulin resistance in humans, and to establish whether varying degrees of insulin resistance in different tissues are involved in the protection from dyslipidemia, fatty liver, and adiponectin suppression we report in this condition.

## Methods

### Clinical studies.

Venous blood was drawn in the fasting state and plasma immediately extracted and stored at –20°C. For oral glucose tolerance testing, 1.75 g glucose/kg bodyweight was given after a 10-hour fast. Insulin, leptin, and adiponectin were assayed using previously described dissociation-enhanced lanthanide fluorescence immunoassays (11). Anti–insulin receptor antibodies were sought using an IP-based assay described previously (49). Other analytes were determined in accredited diagnostic laboratories of referring hospitals. BMI-adjusted normative adiponectin and leptin data were derived from a European ancestry population from the MRC Ely Study cohort (11, 50). Normative data for liver triglyceride content were derived from the most insulin-resistant quartile of sex- and ancestry-matched patients from the Dallas Heart Study for P1(28).

Body composition was measured by Lunar Prodigy DXA (GE Healthcare). Hepatic triglyceride content was assessed by proton MRS (Siemens 3T Verio scanner) (9) and quantified as the ratio of methylene and combined methylene and water signals corrected for spin-spin relaxation and expressed as a weight percentage. De novo lipogenesis was assessed using gas chromatography/mass spectroscopy by measuring the incorporation of deuterium into plasma triglycerides during administration of deuterium-labeled water (5973N GC/MS system; Agilent Technologies). All protocols have been described previously (9).

### Genetic studies.

Exons and at least 100 bp of exon-flanking sequences of the *INSR* gene were sequenced using Sanger sequencing of PCR products using the BigDye Terminator v3.1 Cycle Sequencing Kit according to the manufacturer’s instructions (Applied Biosystems, Life Technologies) and run on ABI 3730 DNA Sequencers (Applied Biosystems). Sequence analysis was performed using the Mutation Surveyor software package v2.3 (SoftGenetics).

For exome sequencing, genomic DNA for P1 and her parents was extracted from blood leukocytes, prepared for capture, and sequenced as described previously (51). Sequencing was performed on the Illumina Genome Analyzer II (index case) or Hi-Seq 2000 platform (parents), as 54 or 75 bp paired-end reads, respectively, and was mapped to the genome reference sequence (NCBI build 37) (51). Variants were called and annotated against Ensembl build 37 (51). Raw exome sequence data from the trio are available from the European Genome-Phenome Archive (https://www.ebi.ac.uk/ega/home; accession numbers EGAN00001001257, EGAN00001001835, and EGAN00001001824).

Exome data for P1 and her parents were merged, and variants with a high posterior probability of being de novo (cutoff value 0.8) were extracted using DeNovoGear v0.5 (https://sourceforge.net/projects/denovogear/) (29). Potential compound heterozygous mutations were also extracted. Variant annotation has previously been described in detail (51). Potentially causal variants identified were reconfirmed by Sanger sequencing. For other patients, exons and exon-intron boundaries of *PIK3R1* were sequenced by Sanger sequencing.

A cohort of 262 patients with severe insulin resistance, assembled as part of a long-running study, was further investigated by Sanger sequencing of the whole *PIK3R1* gene, as well as *PIK3R2*, encoding the p85β subunit, and *PIK3CA*, encoding the p110α catalytic subunit. All primer sequences are available in Supplemental Table 4.

### Cell culture.

Generation and culture of EBV-transformed lymphoblastoid cell lines and dermal fibroblasts have been described previously (52). HEK293-T cells (ATCC) were maintained in DMEM containing 25 mM glucose supplemented with 10% FBS, 2 mM l-glutamine, 100 U/l penicillin, and 100 μg/ml streptomycin (all Sigma-Aldrich). 3T3-L1 preadipocytes (ZenBio) were cultured and differentiated as previously described (52).

### Plasmid constructs and stable cell line generation.

A pSG5 plasmid encoding N-terminal HA-tagged WT human p85α (pSG5.HA.p85α) was a gift from Bart Vanhaesebroeck, University College London. A pLP-LNCX plasmid containing N-terminal FLAG-tagged human p110α (pLP-LNCX.FLAG.p110α) was obtained from Addgene (53). A pCNX2 plasmid containing N-terminal FLAG-tagged human adiponectin coding sequence (pCNX2.FLAG.ADIPOQ) was a gift from Louis Luttrell, Medical University of South Carolina, Charleston, South Carolina (54). Site-directed mutagenesis of the p85α and adiponectin plasmids was performed using primers listed in Supplemental Table 5 and the Quikchange Kit (Agilent, Stratagene), as per the manufacturer’s guidelines, and confirmed by direct nucleotide sequencing (55).

For protein and RNA analysis in PI3K studies, HEK293-T cells were transiently transfected 1 day after seeding into 6-well plates with 1.25 μg/well WT or mutant pSG5.HA.p85α and 1.25 μg/well pLP-LNCX.FLAG.p110α, or 2.5 μg/well pcDNA3.1 empty vector, using Lipofectamine LTX (Invitrogen), as per the manufacturer’s guidelines. For PIP_3_ quantification and PI3K assays, cells were seeded into 10-cm dishes and transfected with 10 μg plasmid. 10% pEGFP.N1 was cotransfected in all wells to assess transfection efficiency (60%–80%). All assays were performed 48 hours after transfection.

3T3-L1 preadipocyte cell lines inducibly expressing WT, Y657X, or R649W mutant p85α were generated by constructing doxycycline-regulated pSLIK-Hygro lentiviral vectors containing either the mutant or WT gene, as described previously (55). For these experiments, *PIK3R1* was amplified from a cDNA library, then subject to site-directed mutagenesis, using protocols described above and primers listed in Supplemental Table 1. VSV-G–pseudotyped lentiviral particles were generated and used to infect 3T3-L1 preadipocytes to generate stable cell lines. Inducibility of p85α overexpression was assessed using immunoblotting for total p85 and quantitative real-time PCR to detect human *PIK3R1*.

### Insulin stimulation studies.

Transfected HEK293-T cells, dermal fibroblasts, or 3T3-L1 preadipocytes were washed once in warm PBS, then serum starved for 16 hours in DMEM supplemented with 0.5% BSA. Cells were stimulated for 5 minutes at 37°C with 100 nM Actrapid insulin (unless indicated otherwise). Cells were lysed in tyrosine kinase lysis buffer (52) or, for PI3K assays, in PI3K lysis buffer (56). To assess the rate of dephosphorylation of AKT, insulin stimulation (as above) was terminated by washing in warm PBS, followed incubation in fresh serum-free medium, for the specified time intervals.

### Gene expression analysis.

Total RNA extraction, reverse transcription, and real-time quantitative PCR were performed as previously described (52). Catalog numbers for inventoried primer/probe mixes are listed in Supplemental Table 6.

### IP and Western blotting.

For IP, cleared cell lysates containing 1 mg protein (for anti-phosphotyrosine IP) or 200 μg protein (for HA or p85 IP) were agitated with primary antibody for 3 hours, then immobilized onto Protein A agarose beads (Sigma-Aldrich). Immunoblotting of 50 μg denatured total cellular protein or 20 μl immunoprecipitate was performed as described previously (52). Sources and concentrations of antibodies are listed in Supplemental Table 7. All blots shown are representative examples of at least 3 independent experiments. Full uncut gels are included in the supplemental material.

### PIP_3_ quantification and PI3K activity assay.

For quantification of cellular PIP_3_, dermal fibroblasts were serum starved overnight, stimulated for 10 minutes with 100 nM insulin, then lysed in 1.5 ml 1M HCl. Lipid extraction, derivatization, and mass spectrometric analysis have been described previously (57).

### Evaluation of ADIPOQ mutant.

Human serum was diluted 10-fold in PBS, incubated with 2× SDS non-reducing Laemmli buffer (Bio-Rad) for 1 hour at room temperature, centrifuged at full speed for 3 minutes, then subjected to SDS-PAGE on a NuPage 4-12% Bis-Tris gradient gel (Invitrogen) in MOPS buffer. In the cellular model, HEK293-T cells were transferred to serum-free medium 48 hours after transfection with 1 μg/well or 2 μg/well WT or mutant pCNX2.FLAG.ADIPOQ. Medium was collected after 24 hours, then incubated at room temperature with nonreducing sample buffer, as above.

### Adipocyte studies.

Neutral lipid staining of 3T3-L1 adipocytes on day 10 post differentiation was performed using Oil Red O as described previously (52).

### Statistics.

Graph preparation and statistical analysis were performed in GraphPad Prism 5.0. Dot plots display mean ± SEM unless indicated otherwise. Differences between more than 2 groups were assessed for statistical significance using 1-way ANOVA, followed by post-hoc Tukey testing. Where indicated, the Holm-Šidák method was used to correct for multiple comparisons between 2 groups. *P* < 0.05 was considered statistically significant.

### Study approval.

Human studies were approved by the UK National Health Service Research Ethics Committee and conducted in accordance with the principles of the Declaration of Helsinki. Participants provided written informed consent for participation in the study and inclusion of photographs in this publication.

## Author contributions

IHD designed and conducted cellular studies, interpreted cellular and genetic data, and wrote the manuscript; PT designed, conducted, and interpreted cellular studies and reviewed the manuscript; AG, NR, AK, and LCF designed, conducted, and interpreted cellular studies; SRu, JC, and MW conducted and interpreted phospholipidomic studies. FP, WB, and AD designed, conducted, and interpreted genetic studies; JH, BM, EM, MD, SZ, and RM performed clinical studies and provided clinical data; AS and SRo undertook liver fat determinations and interpreted data. DBS and SO shared in conception of the study and reviewed the manuscript. RKS and IB conceived of, designed, and led the study and wrote the manuscript.

## Supplementary Material

Supplemental data

## Figures and Tables

**Table 1:**
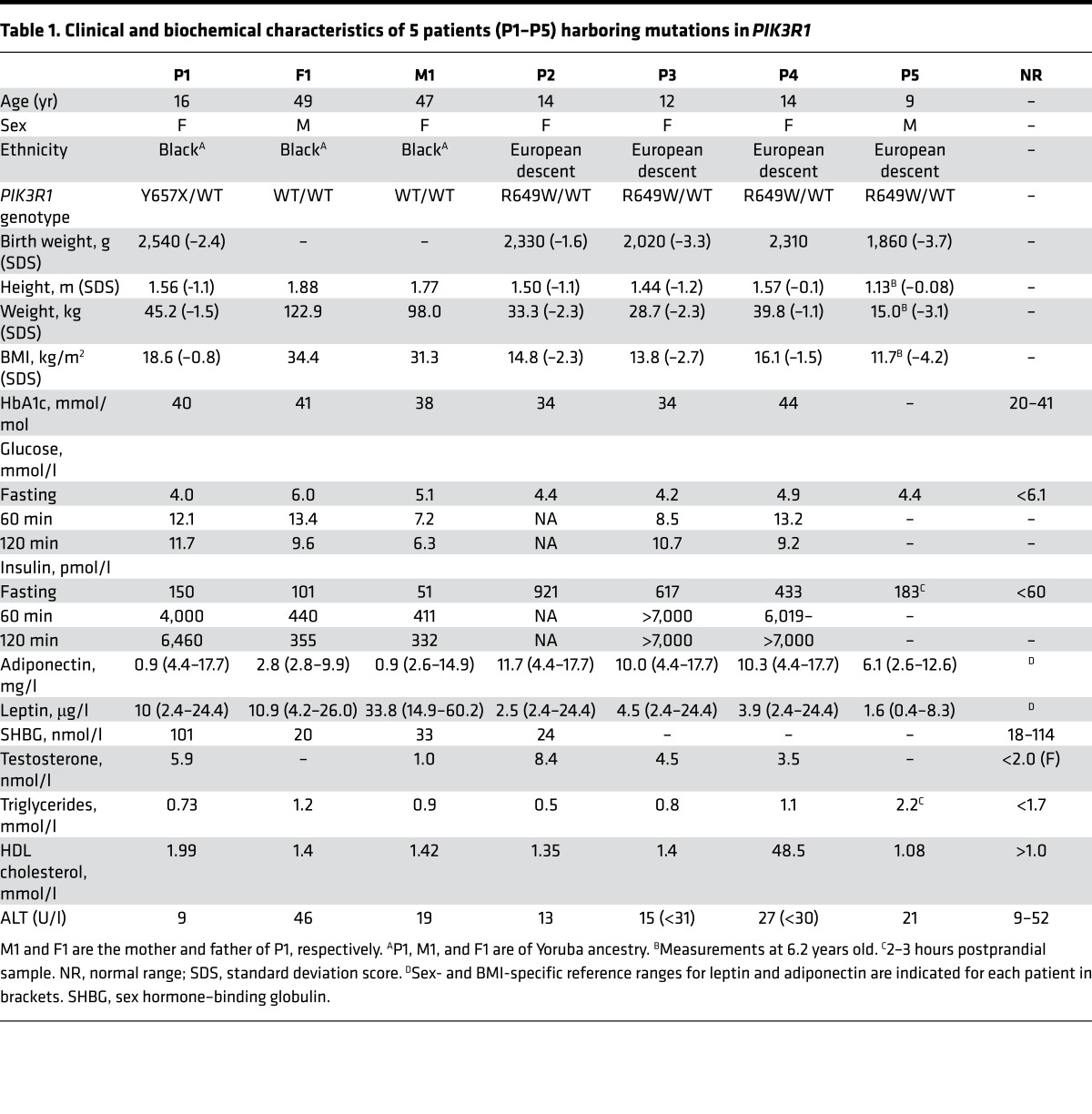
Clinical and biochemical characteristics of 5 patients (P1–P5) harboring mutations in *PIK3R1*
