# Supplementary material for: Insulin resistance uncoupled from dyslipidemia due to C-terminal PIK3R1 mutations
Source: JCI Insight. 2016 Oct 20;1(17):e88766. doi: 10.1172/jci.insight.88766 (PMC5070960; doi:10.1172/jci.insight.88766)

Huang-Doran *et al*

Supplemental Appendix

This appendix has been provided by the authors to give readers additional information about their work.

SUPPLEMENTAL CASE STUDIES 2

SUPPLEMENTAL TABLES 4
Supplemental Table 1. *De novo* mutations found in Patient P1 4
Supplemental Table 2. Rare sequence variants identified in *PIK3R1*, *PIK3R2* and *PIK3CA* in 262 severely insulin resistant probands 5
Supplemental Table 3. Summary of clinical features of 5 probands with *PIK3R1* mutations 6
Supplemental Table 4. Primer sequences for Sanger sequencing of PI3K subunits 7
Supplemental Table 5. Primer sequences for site-directed mutagenesis 9
Supplemental Table 6. PCR primers and probes used for quantitative real-time PCR 10
Supplemental Table 7. Antibodies used for western blotting and immunoprecipitation 11

SUPPLEMENTAL FIGURES 12
Supplemental Figure 1. Expression of type 1A PI3K regulatory subunit genes in EBVLs from Patient 1 12
Supplemental Figure 2. Interaction of mutant p85α with phosphotyrosine residues and p110 within a heterologous expression system. 13
Supplemental Figure 3. AKT dephosphorylation in primary dermal fibroblasts 14
Supplemental Figure 4. PTEN expression and association with p85 in primary dermal fibroblasts. 15
Supplemental Figure 5. Axenfeld-Rieger anomaly in Patient 2 16

REFERENCES 17

SUPPLEMENTAL CASE STUDIES

Patient 2

Patient 2 (P2) is currently 14.5 years old. She was born at term to non-consanguineous parents of European ancestry. Pregnancy was complicated by an amniotic fluid leak and at 30 weeks of gestation intra-uterine growth restriction was noted. Feeding difficulties during infancy were attributed to gastro-oesophageal reflux. Weight remained at or below the 3rd percentile from infancy. Head circumference followed the 10th percentile and length was between the 3rd and 10th percentile. She had a left duplex kidney and recurrent urinary tract infections secondary to grade 4 urinary reflux, eventually requiring ureteric re-implantation surgery at 5 years. There was no history of nephrocalcinosis.

At two years old, dysmorphic features including a triangular face, wide mouth, thin upper lip, an “unusual” nose with prominent veins on the scalp, face and trunk were noted. The ears were prominent, posteriorly-placed and slightly low-set. There was reduced subcutaneous fat on the head, trunk and over the buttocks, but normal fat coverage on the legs and arms.

At six years old, facial dysmorphism was again noted. Investigation at this age revealed normal lipid and immunoglobulin profiles. At 10.5 years onset of puberty was noted with breast bud development.

At 13.5 years, the age at which the detailed biochemical evaluation is reported, she had narrowed facial features, deep set eyes, prognathism, a pointed chin and nose and over-crowded teeth in the upper and lower jaw. There was widespread acanthosis nigricans, most prominent in the nuchal and periumbilical regions and behind the ears. She was very lean with muscular arms and legs, and very little subcutaneous fat over the face and around her hands. Nails were concave. A deepening voice, hirsutism, and an enlarging laryngeal prominence were noted. Breast development had regressed, and primary amenorrhoea was reported. Pelvic ultrasonography showed numerous small, circumferential ovarian follicles. At 13.8 years, bone age was 15 years. Metformin was started.

No visual problems nor refractive error were reported, however ophthalmological assessment revealed Axenfeld-Rieger anomaly with iris hypoplasia, iridogoniodysgenesis, and posterior embryotoxon (Supplemental Figure 5). On gonioscopy of the angle, iris strands associated with redundant peri-umbilical skin were detected. Ocular pressures and nerves were normal.

Her mother was fit and well. Her father was unavailable for assessment but was said to be 160 cm in height, to be of slight build and to have “fine features”, raising the unconfirmed possibility of autosomal dominant inheritance.

Patient 3

Patient 3 (P3) is currently 13 years old, is the second daughter of unrelated Italian parents, and has two healthy siblings. She was born at term after fetal growth was found to be subnormal in the last 2 months of gestation. Birth weight was 2200 g (-2.2 SDS). Facial dysmorphism was noted at

birth, including ocular depression, a prominent nose and generalized paucity of adipose tissue. Subsequent psychomotor development was normal. Investigation including brain MRI, LMNA gene sequencing and fasting plasma insulin were said to be normal in infancy.

On endocrine evaluation at age 12 years, height was 144.1 (-1.1 SDS) and weight 28.7 kg (BMI 13.8). Despite well advanced puberty (Tanner stage 4), menarche had not occurred. On physical examination, generalized reduction of adipose tissue, a triangular face with small chin, thin upper lip, and prominent ears were noted. Skin was pale and dry with prominent veins. Severe acanthosis nigricans was present. She did not show joint hyperextensibility.

Patient 4

Patient 4 (P4) is a girl currently aged 14 years old, born at term to unrelated German parents. Intrauterine growth retardation had been observed ultrasonographically during the pregnancy. At birth facial dysmorphism was noted, and she subsequently exhibited poor linear growth, persisting between the 3rd and 10th centiles for height, however development was otherwise normal. At 8 years old she developed a squint and myopia was diagnosed, with raised intraocular pressure later detected at 11 years old. At 13.5 years old glycosuria was detected during an episode of abdominal pain. Further metabolic investigation at that age showed impaired glucose tolerance with extreme hyperinsulinemia during oral glucose tolerance testing.

At 14 years old menarche had not yet occurred although puberty was well established (Tanner A2 B2 P4). There was generalised paucity of subcutaneous adipose tissue, and facial dysmorphism featuring a triangular face with a prominent forehead, deep set eyes, large and low set ears, a narrow nose and dimpled chin. There was no acanthosis nigricans.

Patient 5

Patient 5 (P5) is currently 9 years old. He is the first son of unrelated Bulgarian parents, and was born following an uncomplicated pregnancy at term. He was admitted to the neonatal unit due to small size and poor feeding. Development progressed normally apart from mild speech and language delay, and delay in tooth eruption.

On examination he had fine facial features with prominent eyes and the appearance of megalocornea and ocular depression, although formal ophthalmology assessment was normal. He had a narrow nasal bridge, prominent columella, low set and prominent ears, wide mouth and extensive dental caries of deciduous teeth. He had normal, thick hair. He had generalized lipoatrophy with prominent veins particularly affecting his face and trunk and giving an ‘aged appearance’. His hands had wrinkled skin. There was no joint hypermobility. He also had a normal renal ultrasound examination and echocardiogram.

SUPPLEMENTAL TABLES

Gene	Mutation	Chr	Position (GRCh37)	ref	alt
PIK3R1	Y657X	5	67592155	T	A
ABCB5	S240F	7	20685419	C	T
MYH10	R690X	17	8424463	G	A

Supplemental Table 1. *De novo* mutations found in Patient P1: Chromosome positions are relative to NCBI build 37 and dbSNP identifiers to version 137. ref refers to the NCBI reference sequence allele, alt refers to the alternate allele. Variants were absent from the 1000 Genomes (release 2012-07-19, 1,092 genomes) – EUR (European ancestry), AMR (from the Americas), AFR (West African ancestry), ASI (East Asian ancestry); NHLBI Exome Variant Server (release ESP6500SI-V2, Apr-2013, 6,250 exomes) – EA (European American ancestry), AA (African American ancestry); 3,781 Genomes from the UK10K Cohorts group (UK10K Cohorts, release 2012-06-02) - all of European ancestry; 409 CoLaus Exomes (ODEX, release Dec-2010) – all of European ancestry and dbSNP (build 137).

Variant	ExAC Allele Frequency	SIFT Prediction (Score)	Polyphen Prediction (Score)	PhD-SNP prediction (Reliability index)
PIK3R1				
R649W		Affect protein function (0.00)	Probably damaging (1.000)	Disease (9)
I133T	0.00002471	Tolerated (0.28)	Benign (0.000)	Neutral (8)
R409Q*	0.000008298	Tolerated (0.10)	Probably damaging (0.958)	Disease (5)
PIK3R2				
P4S	0.004116	Tolerated (0.56)	Benign (0.022)	Neutral (9)
V54M	0.003671	Affect protein function (0.01)	Possibly damaging (0.684)	Neutral (7)
P151S	0.01130	Tolerated (0.92)	Benign (0.000)	Neutral (9)
PIK3CA				
N497S	0.000008284	Tolerated (0.62)	Benign (0.427)	Neutral (8)
M610V	0.00002514	Tolerated (0.44)	Benign (0.000)	Neutral (8)
Y644H		Affect protein function (0.00)	Probably damaging (1.000)	Neutral (1)
R951L		Affect protein function (0.00)	Probably damaging (2.918)	Disease (2)

Supplemental Table 2. Rare sequence variants identified in *PIK3R1*, *PIK3R2* and *PIK3CA* in 262 severely insulin resistant probands: Heterozygous, functional (non-synonymous) single nucleotide variants in *PIK3R1* (encoding p85α, p55α and p50α), *PIK3R2* (encoding p85β) and *PIK3CA* (encoding p110α) identified in 262 severely insulin resistant patients, including *PIK3R1* p.Arg649Trp (R649W, underlined). Where available, allele frequencies were obtained from the Exome Aggregation Consortium (ExAC) (1). Predicted effects of variants on protein function were determined using three online algorithms: SIFT Sequence (Database: UniRef90 2011 Apr, Median conservation of sequences 3.00), Polyphen-2 (v2.2.2) and PhD-SNP on Uniprot ID P27986 (algorithm-specific scores in parentheses) (2-4). Note that the R951L variant in *PIK3CA* did not co-segregate with insulin resistance in the family. The R409Q variant in *PIK3R1* has been previously reported in associated with severe human insulin resistance (5).

Feature	P1<br>Y657X	P2<br>R649W	P3<br>R649W	P4<br>R649W	P5<br>R649W
IUGR	Yes	Yes	Yes	Yes	Yes
Short Stature	Yes	Yes	Yes	Yes	Yes
Hyperextensibility of Joints	No	Yes	No	No	No
Ocular Depression	Yes	Yes	Yes	Yes	Yes
Rieger Anomaly<br>(other opthalmologic problems)	No	Yes (Supp. Figure 6)	No	No<br>(myopia from 8 years; raised intraocular pressure from 11 years)	No
Teething Delay	Not noted	No	Not noted	Not noted	Yes
Facial Dysmorphism	Yes	Yes	Yes	Yes	Yes
Adipose Tissue	Normal	Reduced	Reduced	Yes	Not known
History of recurrent infections	No	No	No	No	No
IgG (NR 5.4-16.1 g/L)	13.7	9.2	7.7	8.0	6.6
IgM (NR 0.5-1.9 g/L)	1.1	1.4	1.1	0.5	1.0
IgA (NR 0.8-2.8 g/L)	1.6	2.1	1.4	1.2	1.3

Supplemental Table 3. Summary of clinical features of 5 probands with *PIK3R1* mutations. NR = Normal Range.

Gene_ _exon	F/R primer	Oligonucleotide sequence
PIK3R1_exon2	F	TGTAAAACGACGGCCAGTTCCTTTGTCCTCTGCTGGAC
	R	AGGAAACAGCTATGACCATTGCACCACACATCAATTCAG
PIK3R1_exon3	F	TGTAAAACGACGGCCAGTCCCTAGGTGAGAAGCAGTGG
	R	AGGAAACAGCTATGACCATTCCCAGTACTTAGCCCCAAAC
PIK3R1_exon4	F	TGTAAAACGACGGCCAGTTAGAGGCCAGATGGTTTTGC
	R	AGGAAACAGCTATGACCATTAACAGGGCAGGGAGAACTG
PIK3R1_exon5	F	TGTAAAACGACGGCCAGTGGAGCAACCCCTACAAGAAG
	R	AGGAAACAGCTATGACCATGGGGGAGGGACAGGTTATTA
PIK3R1_exon6	F	TGTAAAACGACGGCCAGTTGTGTGCTTCTCCCAACAAC
	R	AGGAAACAGCTATGACCATATCTGCACATGCATCTCTGG
PIK3R1_exon7	F	TGTAAAACGACGGCCAGTTTCAGCCCTATGCTTTTCAGA
	R	AGGAAACAGCTATGACCATTCCACAAATGTTGAAACTCG
PIK3R1_exon8	F	TGTAAAACGACGGCCAGTTTGAGCCATGCTCTGAAAGA
	R	AGGAAACAGCTATGACCATCCACAGTTCTCCAGTGCTCA
PIK3R1_exon9	F	TGTAAAACGACGGCCAGTATCTATGTGGGCAGGAGGAA
	R	AGGAAACAGCTATGACCATAAGCCATATTTCCCATCTCG
PIK3R1_exon10	F	TGTAAAACGACGGCCAGTACGGGACCTTTTTGGTACG
	R	AGGAAACAGCTATGACCATCAAAATAGCTGACATGGAAACATC
PIK3R1_exon11	F	TGTAAAACGACGGCCAGTAACCACTACCGGAATGAATCTC
	R	AGGAAACAGCTATGACCATCCCCACCTCATTCGTAAAAAC
PIK3R1_exon12_13	F	TGTAAAACGACGGCCAGTTGCCAGAGGGAACAGAAAAC
	R	AGGAAACAGCTATGACCATTCCACGTGATCATTCAAAGC
PIK3R1_exon13_14	F	TGTAAAACGACGGCCAGTAGGAAAACTGCTGGGAAACC
	R	AGGAAACAGCTATGACCATAAAGCAAGTCATGCATTTTCC
PIK3R1_exon15	F	TGTAAAACGACGGCCAGTTGGAGGCTGAATACCAGGAG
	R	AGGAAACAGCTATGACCATAATCTTTGCCCCCAAAACTC
PIK3R1_exon16	F	TGTAAAACGACGGCCAGTTTACCTACCCAAGGCACTCG
	R	AGGAAACAGCTATGACCATGCCTTAGGCTGCATGTCTTC
PIK3R2_exon2	F	TGTAAAACGACGGCCAGTGCCTGTGACTGCTGGAGATAG
	R	AGGAAACAGCTATGACCATGGACAGAAGAGGAGACCAAGG
PIK3R2_exon3	F	TGTAAAACGACGGCCAGTGAGGTTCAGCCAGCAAAGAG
	R	AGGAAACAGCTATGACCATTTTGCTGCCCAGAGATAAGG
PIK3R2_exon4_5	F	TGTAAAACGACGGCCAGTGCAGCAAAGGGAGACTGC
	R	AGGAAACAGCTATGACCATGGAAGCGCAGCGTGAG
PIK3R2_exon6	F	TGTAAAACGACGGCCAGTCGGCATTAAGAGCTTCCTGC
	R	AGGAAACAGCTATGACCATTTCTCACTCTGGACCCCATC
PIK3R2_exon7_8	F	TGTAAAACGACGGCCAGTAAGCGCACGCACAATCCT
	R	AGGAAACAGCTATGACCATCTCCTCCCTGTGGGAACAT
PIK3R2_exon9	F	TGTAAAACGACGGCCAGTTGCAGGATGCTGAGTGGTAC
	R	AGGAAACAGCTATGACCATGTAGAGACGGGGTTTCACCA
PIK3R2_exon10	F	TGTAAAACGACGGCCAGTCTGGACAACAGAGCAGCAAG
	R	AGGAAACAGCTATGACCATCTGGGAGGTCCGTGTGTACT
PIK3R2_exon11	F	TGTAAAACGACGGCCAGTCAGTACAATGCCAAGCTGGA
	R	AGGAAACAGCTATGACCATCTCTGTGACCTGCAACTGGA
PIK3R2_exon12	F	TGTAAAACGACGGCCAGTGACCAGCTTGAGCAGTCTCC
	R	AGGAAACAGCTATGACCATGATTACAGGCCTGAGCCATC
PIK3R2_exon13	F	TGTAAAACGACGGCCAGTTGAGGTAGGAGGGTCACCTG
	R	AGGAAACAGCTATGACCATGACACTCCCATTGCACACAC
PIK3R2_exon14	F	TGTAAAACGACGGCCAGTAGCCTCTGGTGATGATGAGG
	R	AGGAAACAGCTATGACCATCAACTTTTCCTCCCTGGTGA
PIK3R2_exon15	F	TGTAAAACGACGGCCAGTTCACCAGGGAGGAAAAGTTG
	R	AGGAAACAGCTATGACCATAAGCTTTCCCCGTTCCAG
PIK3R2_exon16	F	TGTAAAACGACGGCCAGTCCCTCCCAGAGCTCTCATTG
	R	AGGAAACAGCTATGACCATAGGGTGAGAGAACCCCAAAC

Cont.

Gene _exon	F/R primer	Oligonucleotide sequence
PIK3CA_exon2A	F	TGTAAAACGACGGCCAGTTCAAGTCAAACTATGGAAAATGAG
	R	AGGAAACAGCTATGACCATTTCTTCACGGTTGCCTACTG
PIK3CA_exon2B	F	TGTAAAACGACGGCCAGTGTTTCTGCTTTGGGACAACC
	R	AGGAAACAGCTATGACCATGATTACGAAGGTATTGGTTTAGACAG
PIK3CA_exon3	F	TGTAAAACGACGGCCAGTTCTACAGAGTTCCCTGTTTGC
	R	AGGAAACAGCTATGACCATTATAAGCAGTCCCTGCCTTC
PIK3CA_exon4	F	TGTAAAACGACGGCCAGTGCAGCCCGCTCAGATATAAAC
	R	AGGAAACAGCTATGACCATCTGGGCGAGAGTGAGATTCC
PIK3CA_exon5	F	TGTAAAACGACGGCCAGTGATTGATCTTGTGCTTCAACG
	R	AGGAAACAGCTATGACCATGGTCAACAGATTACTGTATAGTGCAAG
PIK3CA_exon6	F	TGTAAAACGACGGCCAGTTCTGAACAAAAATTCCGTGGT
	R	AGGAAACAGCTATGACCATAACTCCGACTTCGTGATCCA
PIK3CA_exon7	F	TGTAAAACGACGGCCAGTTGCCTTTTCCAATCAATCTC
	R	AGGAAACAGCTATGACCATAATTCCTGAAGCTCTCCCAAG
PIK3CA_exon8	F	TGTAAAACGACGGCCAGTTGAATTTTCCTTTTGGGGAAG
	R	AGGAAACAGCTATGACCATTTGGCATGCTCTTCAATCAC
PIK3CA_exon9	F	TGTAAAACGACGGCCAGTTTTGCTGAACCCTATTGGTG
	R	AGGAAACAGCTATGACCATTGAATGAAGGCAAGCTAGGG
PIK3CA_exon10	F	TGTAAAACGACGGCCAGTGATTGGTTCTTTCCTGTCTCTG
	R	AGGAAACAGCTATGACCATTGTAAATTGTGCTTTATTTATTCC
PIK3CA_exon11	F	TGTAAAACGACGGCCAGTTCCATTTGCATTTTCCTTTTG
	R	AGGAAACAGCTATGACCATTTTCATTTATTTATGTGGACTTTCTGA
PIK3CA_exon12	F	TGTAAAACGACGGCCAGTAAAATCAGAAGTTAAGGCAGTGTTTT
	R	AGGAAACAGCTATGACCATGCATGGCCGATCTAAAGAAA
PIK3CA_exon13	F	TGTAAAACGACGGCCAGTATTCTAGATCCATACAACTTCCTTTT
	R	AGGAAACAGCTATGACCATGCTCATCACTGGTACAAAATAC
PIK3CA_exon14	F	TGTAAAACGACGGCCAGTCTGAAACTCATGGTGGTTTTGT
	R	AGGAAACAGCTATGACCATCAGCTGAGAGGCAGTGGAAC
PIK3CA_exon15	F	TGTAAAACGACGGCCAGTAGTGCTGCCAGTCTTGCTTC
	R	AGGAAACAGCTATGACCATTCTTATTTTGAGGGTAGGAGAATG
PIK3CA_exon16	F	TGTAAAACGACGGCCAGTGGATTCCTAAATAAAAATTGAGGTGA
	R	AGGAAACAGCTATGACCATTGCATATTTCAAAGGTCAAGACA
PIK3CA_exon17	F	TGTAAAACGACGGCCAGTTTGCTTTCCTGAAGTTTCTTTTG
	R	AGGAAACAGCTATGACCATCCAGAGGCAGTAGCAGAGTG
PIK3CA_exon18	F	TGTAAAACGACGGCCAGTGAAAATTAAAATTGGGGAAAGG
	R	AGGAAACAGCTATGACCATTTTCTCTGTTCTAACTCAGAGGAATAC
PIK3CA_exon19	F	TGTAAAACGACGGCCAGTGGAAACTTGCACCCTGTTTTC
	R	AGGAAACAGCTATGACCATATTGCTCGAGCTCACCTCTC
PIK3CA_exon20	F	TGTAAAACGACGGCCAGTTGGGAATTGGAGATCGTCAC
	R	AGGAAACAGCTATGACCATGCTGGTTTCAATTCCTGAGC
PIK3CA_exon21	F	TGTAAAACGACGGCCAGTGCTTTGTCTACGAAAGCCTCT
	R	AGGAAACAGCTATGACCATTTCTTGCTGTAAATTCTAATGCTG

Supplemental Table 4. Primer sequences for Sanger sequencing of PI3K subunits.
Abbreviations: F, forward primer; R, reverse primer.

Gene	Experiment	F/R primer	Oligonucleotide sequence	Length, bps	T_m, °C	G/C content, %
PIK3R1	Gene amplification	F	ATGAGTGCTGAGGGGTAC	18	58.1	55.6
		R	TCATCGCCTCTGCTGT	16	59.7	56.3
PIK3R1	Y657X SDM	F	GTAAACAGGGCTGCTAAGCCTGCTCTGTAGTG	32	82.2	53.1
		R	CACTACAGAGCAGGCTTAGCAGCCCTGTTTAC			
PIK3R1	Y657X SDM (for stable over-expression)	F	TACAGAGCAGGCCTAGCAGCCCTGTTTACTGC	32	77.7	56.3
		R	GCAGTAAACAGGGCTGCTAGGCCTGCTCTGTA			
PIK3R1	R649W SDM	F	CTGTTTACTGCTCTCCCAGACAAGAAAAGTGCCAT	35	76.5	45.7
		R	ATGGCACTTTTCTTGTCTGGGAGAGCACTAAACAG			
ADIPOQ	D231A	F	GCTGATAATGACAATGCCTCCACCTTCACAGGC	33	82.2	51.5
		R	GCCTGTGAAGGTGGAGGCATTGTCATTATCAGC			

Supplemental Table 5. Primer sequences for site-directed mutagenesis: Oligonucleotide primers for site-directed mutagenesis were designed according to the criteria listed in the Quikchange site-directed mutagenesis kit (Stratagene) and obtained from Sigma-Aldrich. Abbreviations: F, forward primer; R, reverse primer; bps, base pairs; Tm, melting temperature; G/C, guanine/cytosine; SDM, site-directed mutagenesis.

Gene Name	Species	Catalogue Number	Sequence
PIK3R1	Human	Hs00236128_m1	N/A
PIK3R2	Human	Hs00178181_m1	N/A
PIK3R3	Human	Hs01103591_m1	N/A
36B4	Human	N/A	Forward: GCAGATCCGCATGTCCCTT<br>Reverse: TGTTTTCCAGGTGCCCTCG<br>Probe: [6FAM]AAGCTGTGGTGCTGATGG[TAMRA]

Supplemental Table 6. PCR primers and probes used for quantitative real-time PCR: Pre-made primer/probe stocks were obtrained from Applied Biosystems (catalogue numbers indicated). Human *36B4* was used as a housekeeping gene; individual former/reverse primer and Taqman probe sequences are listed (generated by Sigma-Aldrich).

Target Protein	Species	Application	Company	Product ID	Dilution factor
AKT	Rabbit	WB	Cell Signalling Technology, Beverly, MA	9272	1000
Calnexin	Rabbit	WB	Abcam, Cambridge, UK	ab13504	1000
ERK1/2	Rabbit	WB	Abcam, Cambridge, UK	ab7942	1000
α tubulin	Mouse	WB	Abcam, Cambridge, UK	ab11303	1000
β actin	Rabbit	WB	Cell Signalling Technology, Beverly, MA	4967	1000
HA-tag (C29F4)	Rabbit	WB/IP	Cell Signalling Technology, Beverly, MA	3724	1000
IRS1	Rabbit	IP	Cell Signalling Technology, Beverly, MA	2382	N/A
Myc-tag (4A6)	Mouse	WB	Santa Cruz Biotechnology, Santa Cruz, CA	05-724	1000
p110α	Mouse	WB	BD Biosciences, San Jose, CA	611398	1000
p110β	Rabbit	WB	Cell Signalling Technology, Beverly, MA	3011S	1000
p85α	Mouse	WB	Gift from Dr L. Foukas (University College London, UK) [Cell Sciences, Canton, MA]	[CMP021]	1000
p85β	Mouse	WB	Gift from Dr L. Foukas (University College London, UK) [Abcam, Cambridge, UK]	[ab28356]	500
Phospho-AKT (Ser473/474)	Rabbit	WB	Cell Signalling Technology, Beverly, MA	9271S	1000
Phospho-AKT (Thr308)	Rabbit	WB	Cell Signalling Technology, Beverly MA	9275	1000
Total AKT	Mouse	WB	Cell Signalling Technology, Beverly, MA	2920	1000
Phospho-p42/44 ERK1/2 (Thr202/Tyr204) (E10)	Rabbit	WB	Cell Signalling Technology, Beverly, MA	9106S	1000
Phosphotyrosine (4G10 Platinum)	Mouse	IP	Millipore, Billerica, MA	05-1050	N/A
PTEN	Mouse	WB/IP	Santa Cruz Biotechnology, Santa Cruz, CA	sc7974	1000
Total p85 "lightening"	Rabbit	WB	Gift from Professor K Siddle (University of Cambridge, UK)	Ref (6)	5000
Rabbit IgG (H+L) HRP-linked	Donkey	WB	Thermo-Scienfic/Pierce, Waltham, MA	31458	5000
Mouse IgG HRP-linked	Goat	WB	Cell Signalling Technology, Beverly, MA	7076	2500

Supplemental Table 7. Antibodies used for western blotting (WB) and immunoprecipitation (IP).

SUPPLEMENTAL FIGURES

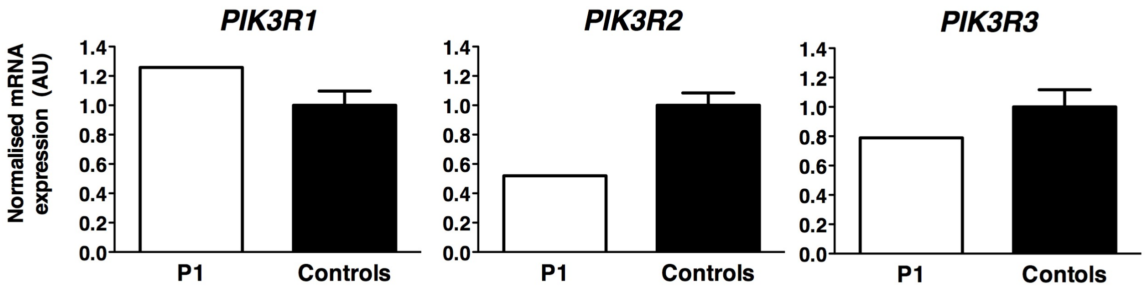

Supplemental Figure 1. Expression of type 1A PI3K regulatory subunit genes in EBVLs from Patient 1: mRNA expression of *PIK3R1*, *PIK3R2* and *PIK3R3* in EBV-transformed lymphoblastoid cells (EBVLs) from Patient 1 (white bars) and three healthy control subjects (black bars), determined by quantitative real-time PCR. Data represent mean +/- SEM.

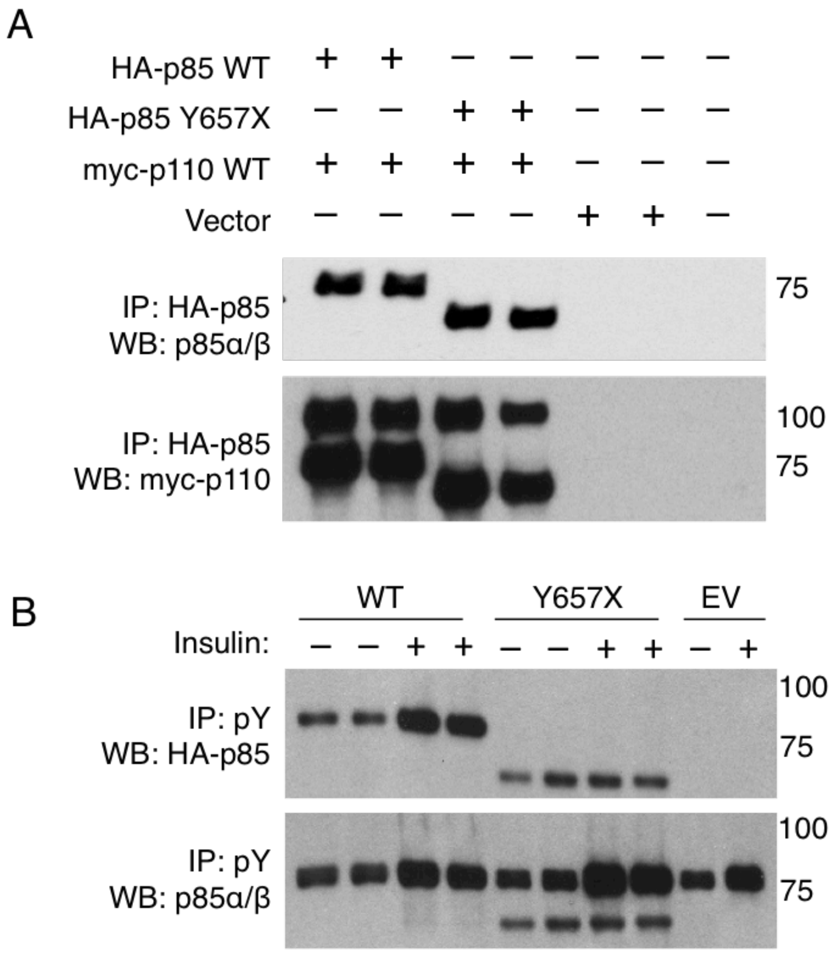

Supplemental Figure 2. Interaction of mutant p85α with phosphotyrosine residues and p110 within a heterologous expression system: (A) Anti-HA immunoprecipitates of HEK293-T cells transiently overexpressing HA-tagged WT or mutant p85α and myc-tagged WT p110α subject to western blotting first against HA (upper panel) and subsequently against myc (lower panel, upper bands; lower bands represent residual signal from the anti-HA antibodies). (B) Western blot of HA-tagged p85α (upper panel) and total p85 (endogenous and recombinant; lower panel) in phosphotyrosine immunoprecipitates of lysates from HEK293-T cells transiently overexpressing HA-tagged wild type or mutant p85a and myc-tagged p110a (or empty vector), treated with PBS or 100nM insulin for 5 minutes. Representative blots shown from three independent experiments.

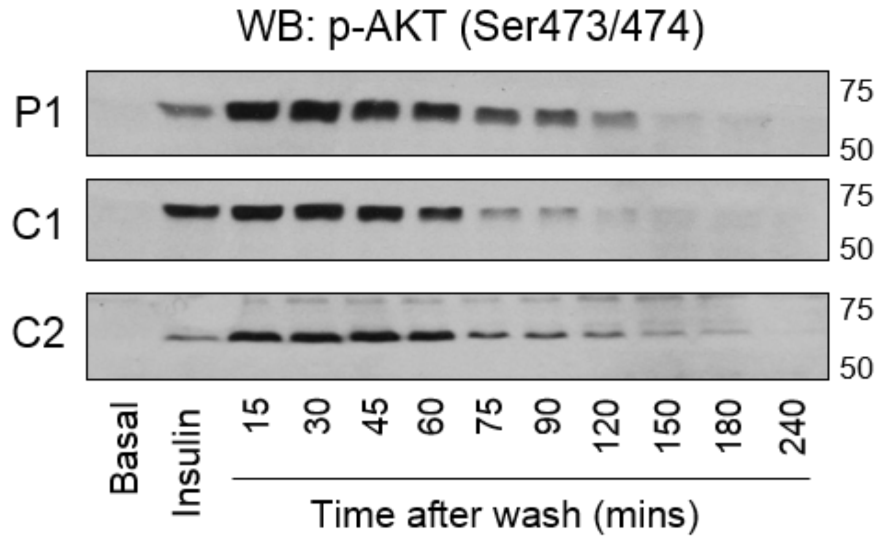

Supplemental Figure 3. AKT dephosphorylation in primary dermal fibroblasts: Western blot of phosphorylated AKT at serine 473/474 in dermal fibroblasts from P1 and two healthy controls (C1, 2) serum-starved overnight, stimulated with 10nM insulin for 5 minutes, washed twice in warm PBS then incubated at 37°C for the times indicated. Representative example of two independent experiments.

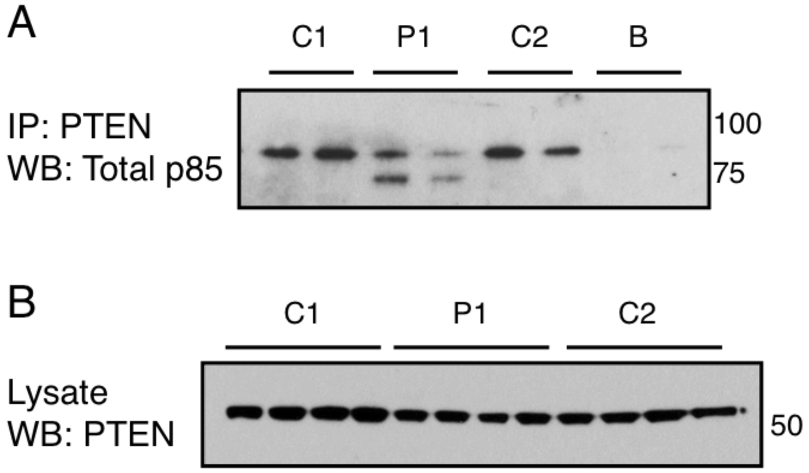

Supplemental Figure 4. PTEN expression and association with p85 in primary dermal fibroblasts: (A) Western blot of total p85 in anti-PTEN immunoprecipitates from dermal fibroblasts of P1 and two healthy controls (C1, C2). Lane marked "*B*" indicates beads only control, without PTEN antibody. (B) Western blot of PTEN in fibrobrast lysates from P1 and two healthy controls (C1, C2).

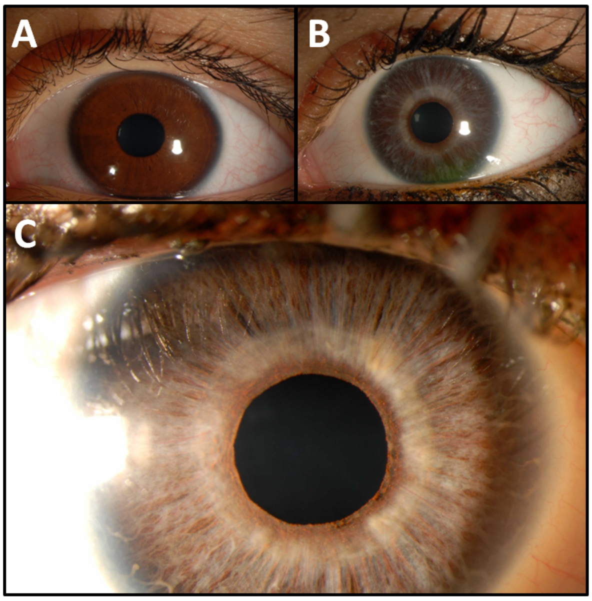

Supplemental Figure 5: Axenfeld-Rieger anomaly in Patient 2: (A) Normal eye for comparison. (B, C). Ocular appearance of P2 showing missing pigment layer and visible blood vessels, exposed sphincter muscle seen as a grey rim around the pupil, and the posterior embryotoxon seen as a faint grey rim around the edge of the cornea.

REFERENCES

1. Exome Aggregation Consortium (ExAC), Cambridge, MA (URL: http://exac.broadinstitute.org).
2. Kumar P, Henikoff S, Ng PC. Predicting the effects of coding non-synonymous variants on protein function using the SIFT algorithm. *Nat Protoc*. 2009;4(7):1073-81.
3. Adzhubei IA, Schmidt S, Peshkin L, Ramensky VE, Gerasimova A, Bork P, Kondrashov AS, Sunyaev SR. A method and server for predicting damaging missense mutations. *Nat Methods* 2010;7(4):248-249.
4. Capriotti E, Calabrese R, Casadio R. Predicting the insurgence of human genetic diseases associated to single point protein mutations with support vector machines and evolutionary information. *Bioinformatics*. 2006;22:2729-2734.
5. Baynes KC, Beeton CA, Panayotou G, Stein R, Soos M, Hansen T, Simpson H, O'Rahilly S, Shepherd PR, Whitehead JP. Natural variants of human p85 alpha phosphoinositide 3-kinase in severe insulin resistance: a novel variant with impaired insulin-stimulated lipid kinase activity. *Diabetologia*. 2000;43(3):321-31.
6. Navé BT, Haigh RJ, Hayward AC, Siddle K, Shepherd PR. Compartment-specific regulation of phosphoinositide 3-kinase by platelet-derived growth factor and insulin in 3T3-L1 adipocytes. *Biochem J.* 1996;318 (Pt 1):55-60.

Huang-Doran et al.

Insulin resistance uncoupled from dyslipidemia in humans with C-terminal mutations in PIK3R1

Full Unedited Gels

Full unedited gels for Figure 2c

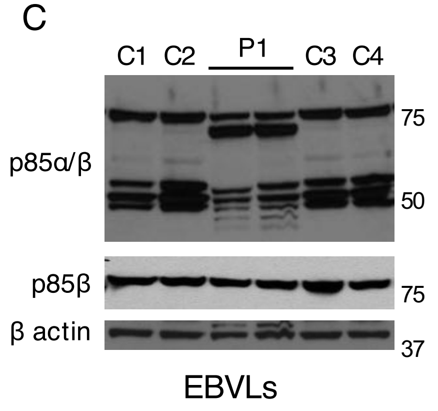

Fig 2c. Full-length and truncated p85α, p55α and p50, and full-length p85β, in EBV-transformed lymphoblastoid cells (EBVLs) from P1 and healthy controls (C1- 4), assessed by western blotting for total p85 (p85α/β) or p85β alone.

p85α/β

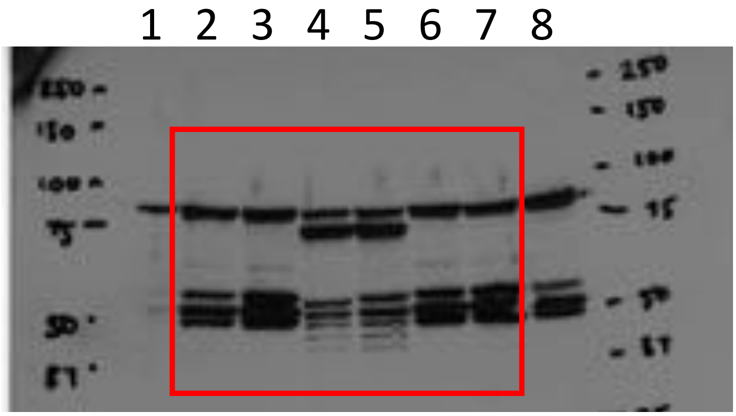

1 = Positive control (HA-p85)
2 = C0158
3 = C0146
4 = P0621
5 = P0621
6 = C0085
7 = C0039
8 = C0212

B actin

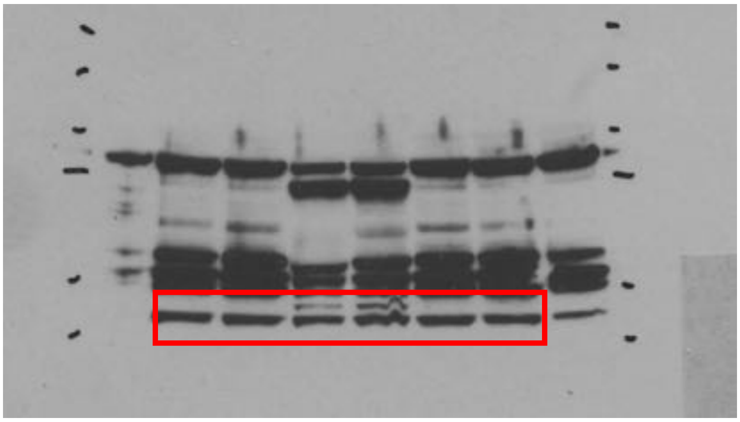

p85β

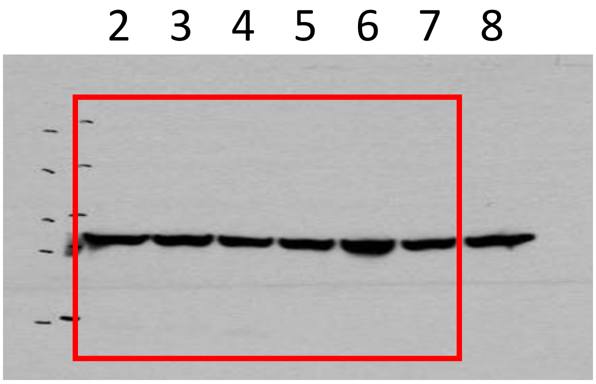

2 = C0158
3 = C0146
4 = P0621
5 = P0621
6 = C0085
7 = C0039
8 = C0212

ID: PI3K_008

Full unedited gels for Figure 2d

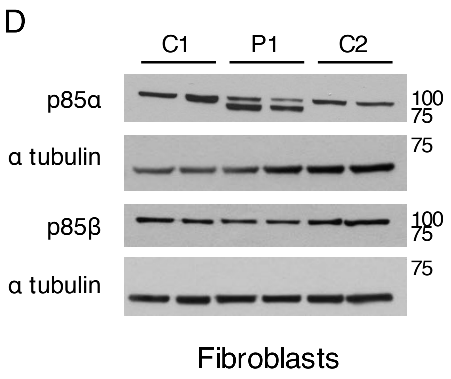

Fig 2d. Representative western blot of p85α or p85β in dermal fibroblasts from P1 and healthy controls (C1, C2).

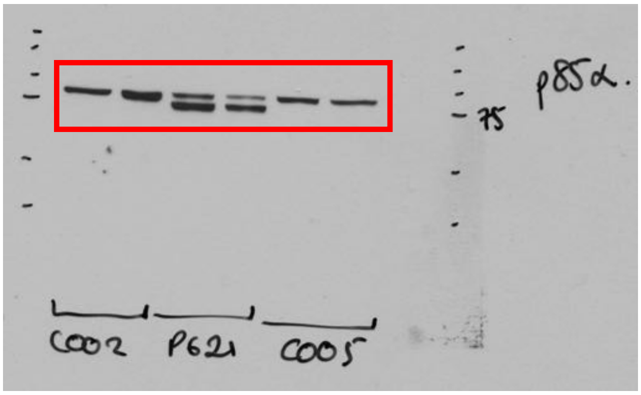

p85α

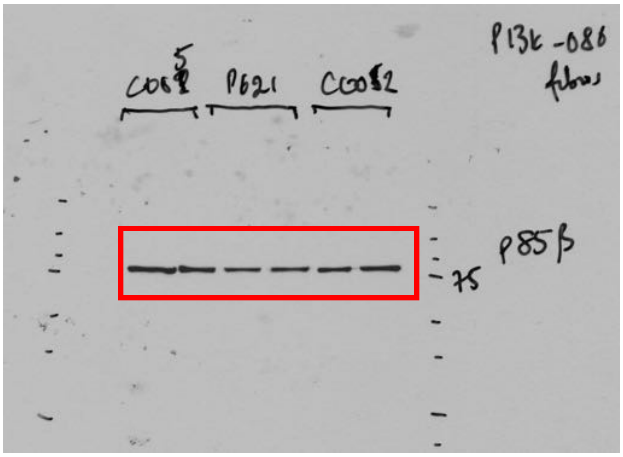

p85β

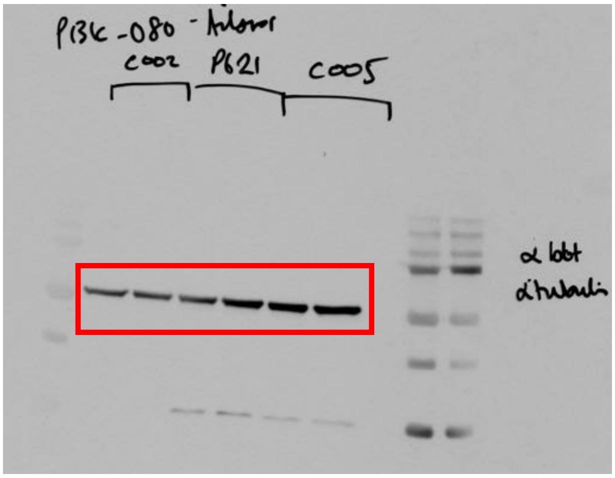

α tubulin

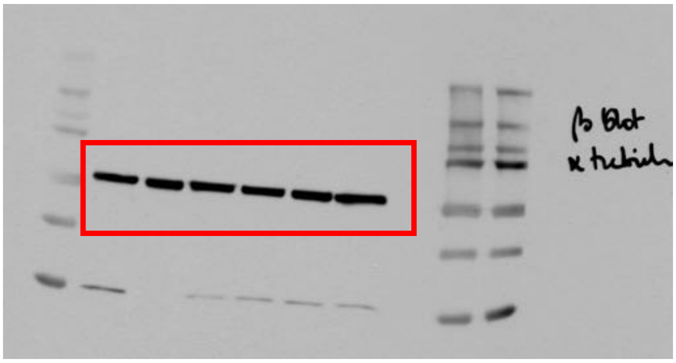

α tubulin

ID: PI3K_080

Full unedited gels for Figure 2f

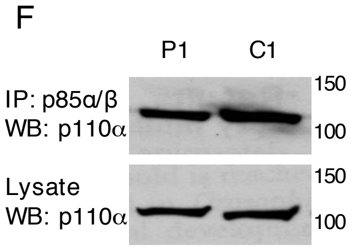

Fig 2f. Western blot of p110α after immunoprecipitation of total p85 in primary dermal fibroblast lysates.

Long exposure

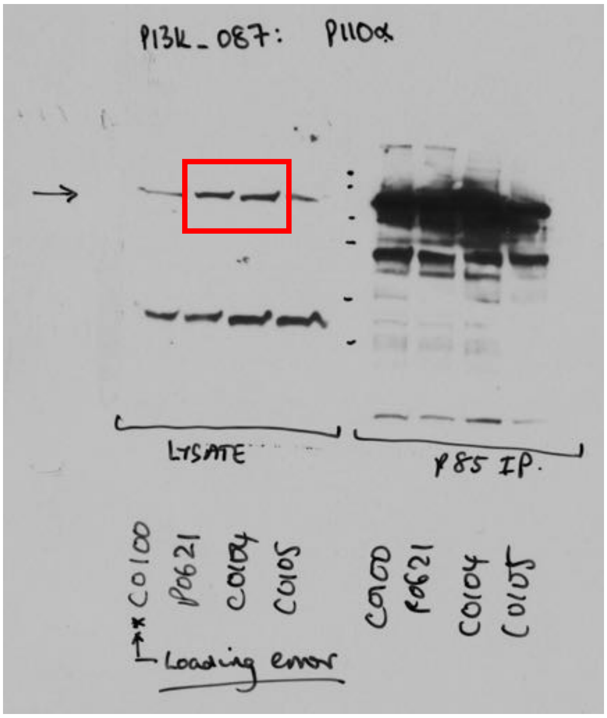

WB: p110α

Short exposure

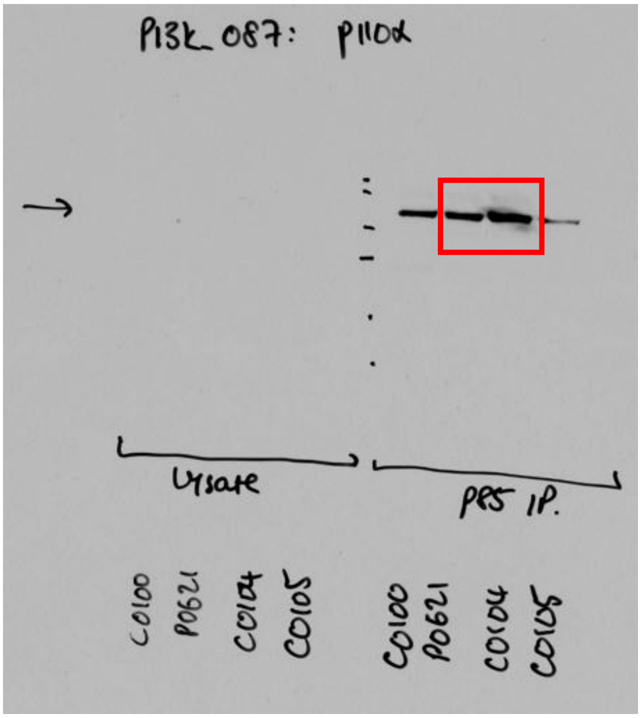

WB: p110α

ID: PI3K_087

Full unedited gels for Figure 2g

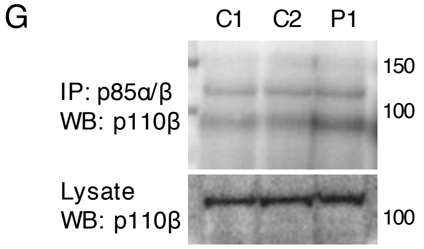

Fig 2g. Western blot of p110β after immunoprecipitation of total p85 in primary dermal fibroblast lysates.

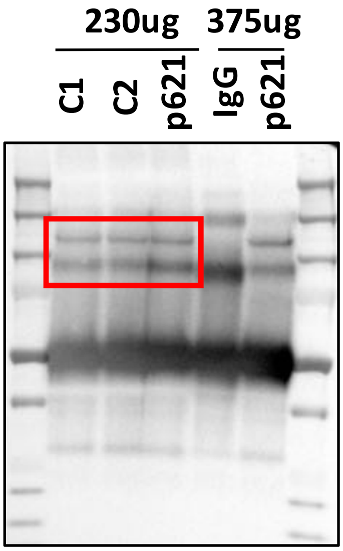

IP: p85α/β
WB: p110β

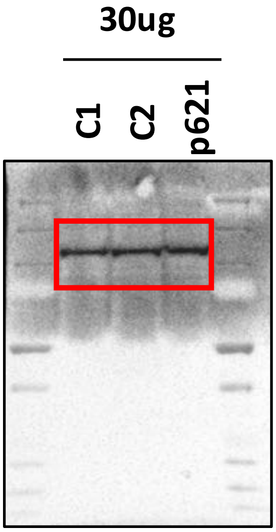

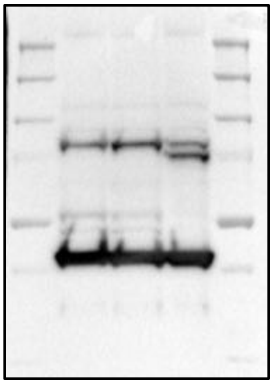

Lysate
WB: p110β

Lysate
WB: β actin (p85)

Full unedited gels for Figure 3a

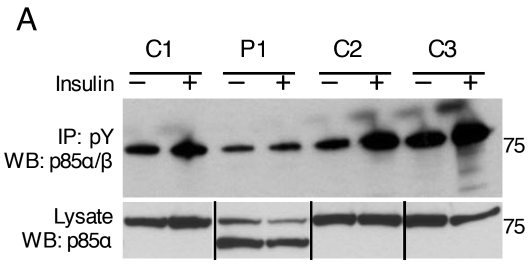

Fig 3a. Western blot of total p85 (p85α/β) in phosphotyrosine immunoprecipitates or total lysates from dermal fibroblasts from P1 and healthy controls (C1-3) treated with PBS or 100nM insulin. Representative blots with quantified data from three independent experiments normalised to mean baseline intensity. Lane order in image of lysate blot adjusted to reflect that of the immunoprecipitate blot.

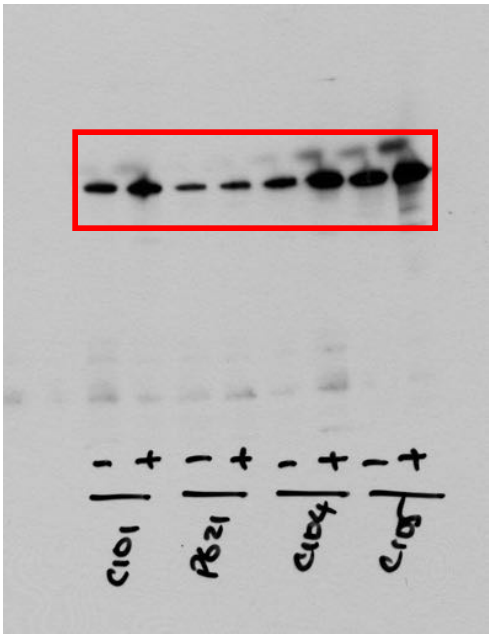

IP: pY
WB: total p85

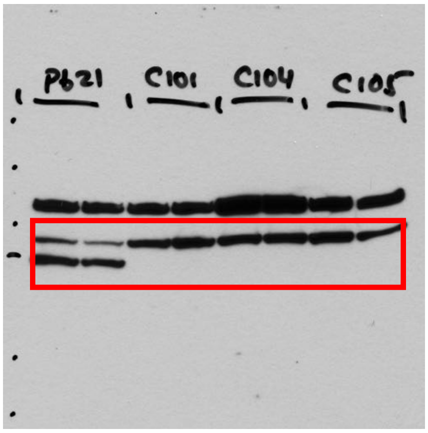

IP: Lysate
WB: p85α

ID: PI3K_089

Full unedited gels for Figure 3b

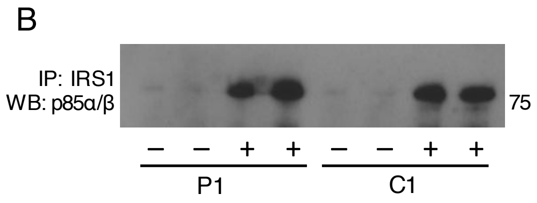

Fig 3b. Western blot of total p85 (p85α/β) in phospho-IRS1 immunoprecipitates from patient and control fibroblasts after treatment with PBS or insulin. Representative blot with quantified data from three independent experiments normalised to mean baseline intensity across control cell lines.

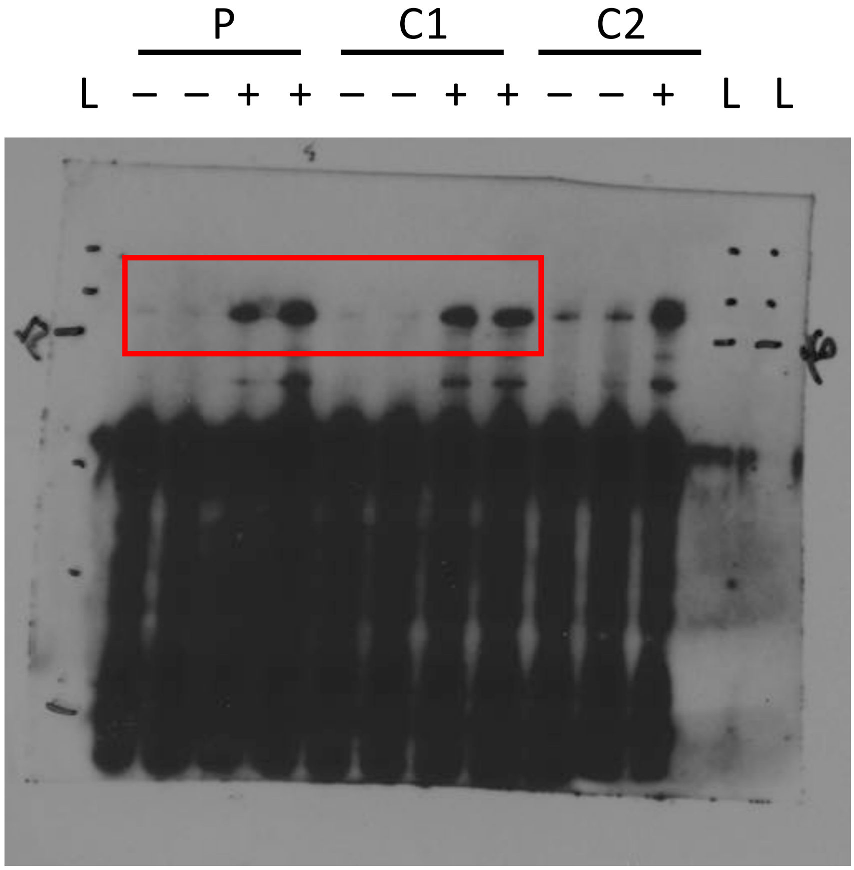

IP: IRS1
WB: total p85

ID: PI3K_116

Full unedited gels for Figure 3d

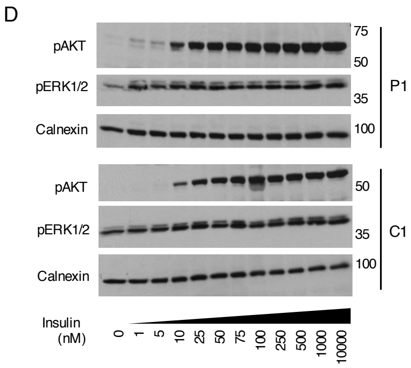

Fig 3d. Western blot of phosphorylated AKT1/2 (Ser473/474) and ERK1/2 (Thr202/Tyr204) in dermal fibroblasts from P1 and healthy controls (C1,2) after stimulation with a range of insulin doses.

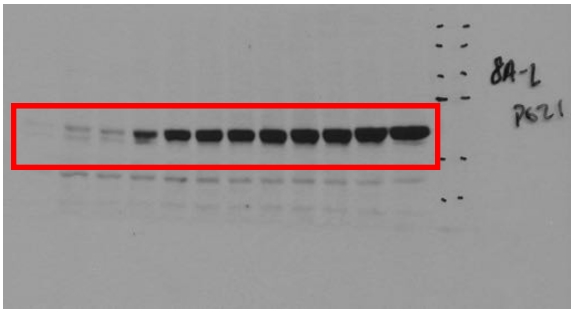

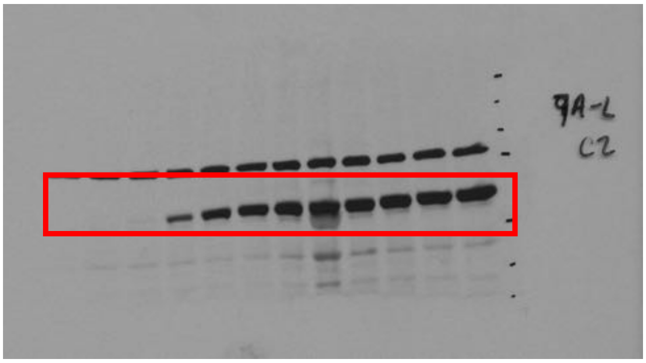

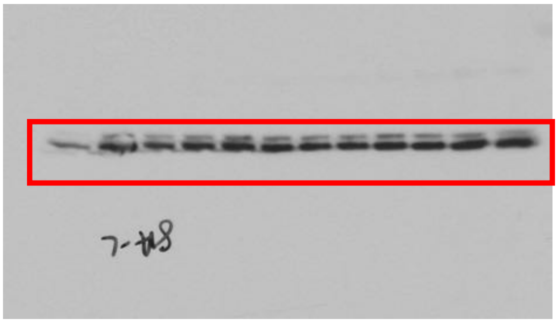

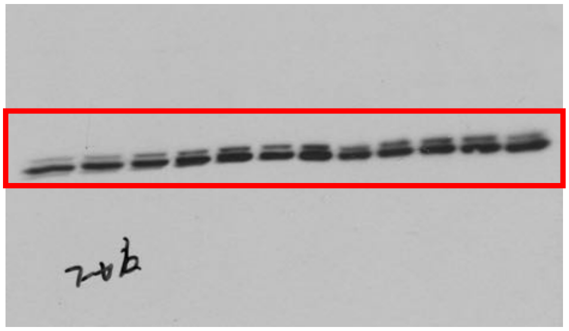

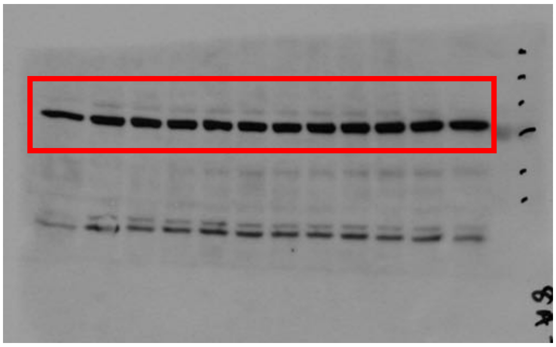

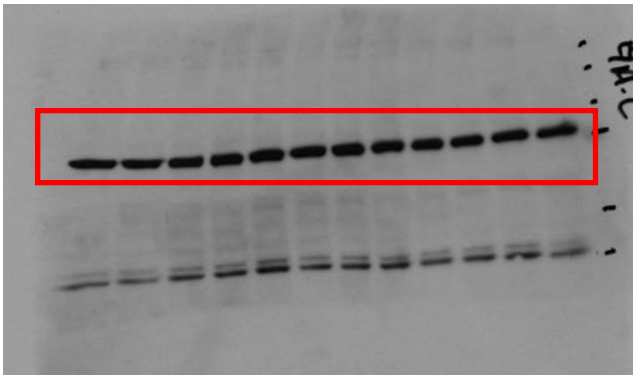

ID: PI3K_064

Full unedited gels for Figure 4a

A

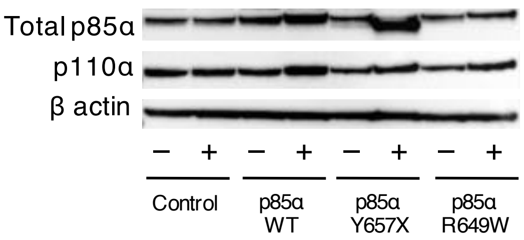

Fig 4a. Total p85 and p110α expression in preadipocyte lysates 72 hours after treatment with or without doxycycline to induce expression of WT or mutant p85α. β actin was assessed as a loading control.

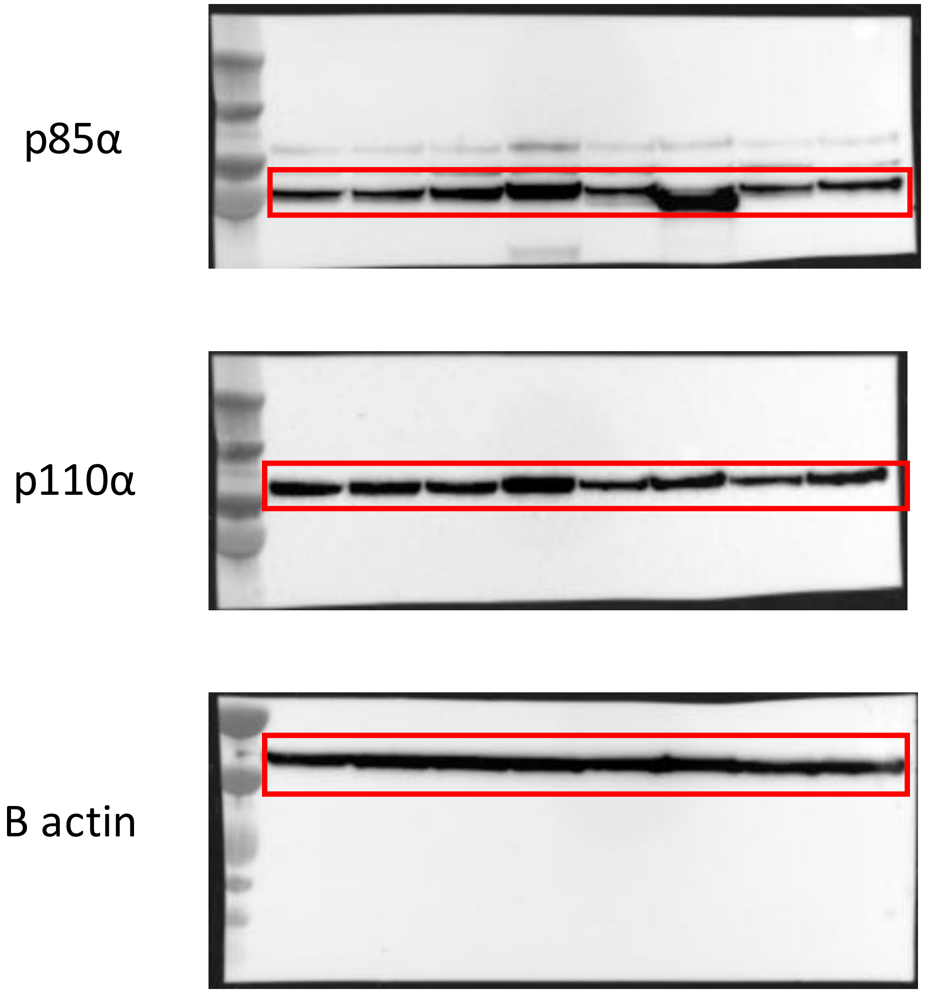

Full unedited gels for Figure 4c

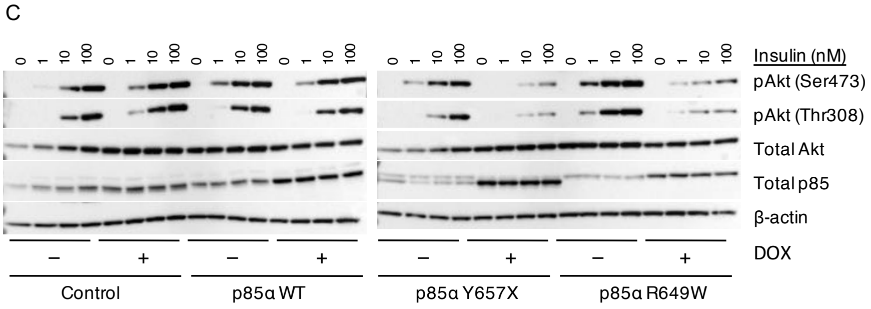

Fig 4c. Insulin-induced phosphorylation of AKT at Ser473/474 and Thr308 in preadipocytes infected with wild type and mutant p85α lentivirus, with or without doxycycline treatment, after stimulation with 1, 10 or 100nM insulin for 10 minutes.

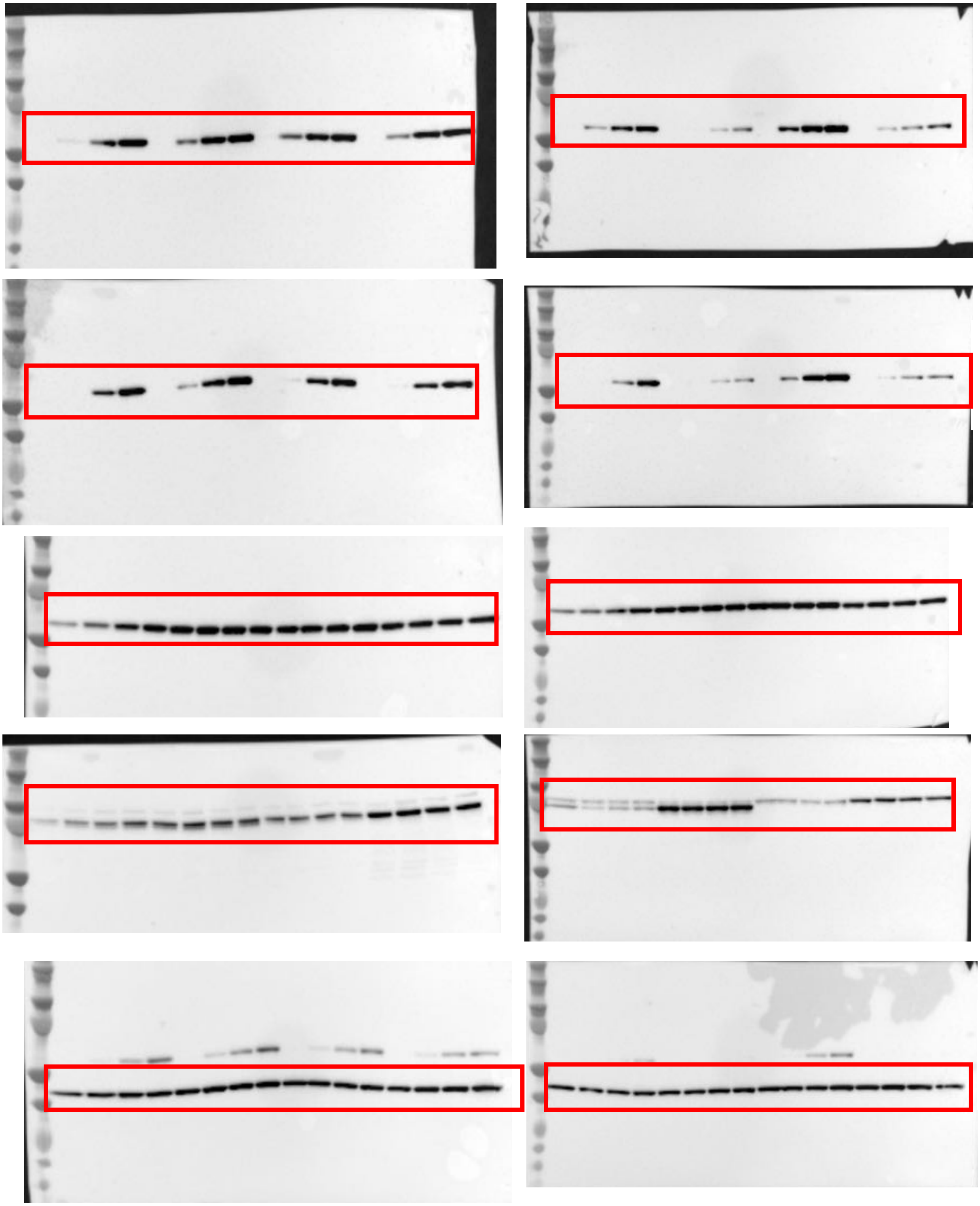

pAkt (Ser473)

pAkt (Thr308)

Total Akt

Total p85

β-actin (Thr308)

Full unedited gel for Figure 5c

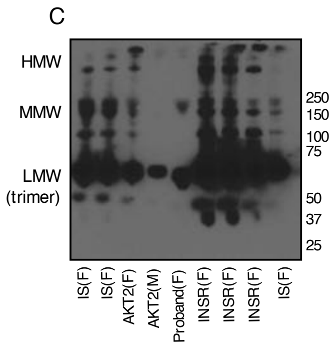

Fig 5c. Representative western blots of adiponectin in serum from P1, insulin resistant controls with mutations in *INSR* or *AKT2*, and insulin sensitive controls (IS), after non-denaturing, non-reducing SDS-PAGE. Low (LMW), medium (MMW) and high (HMW) molecular weight adiponectin complexes indicated. F/M denotes female/male.

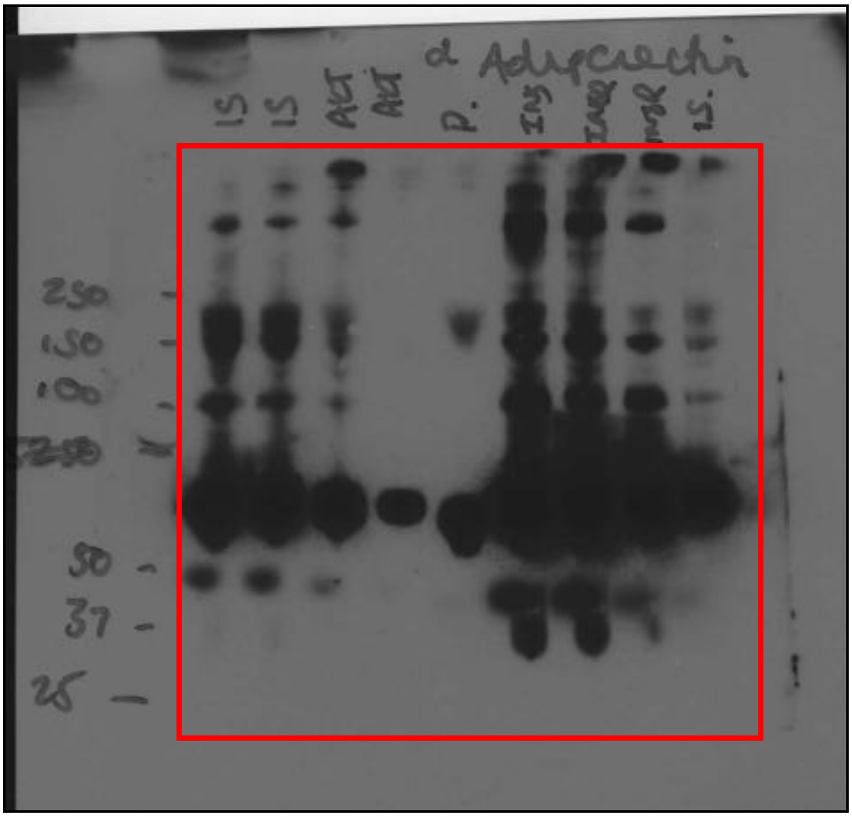

Full unedited gel for Figure 5d

D

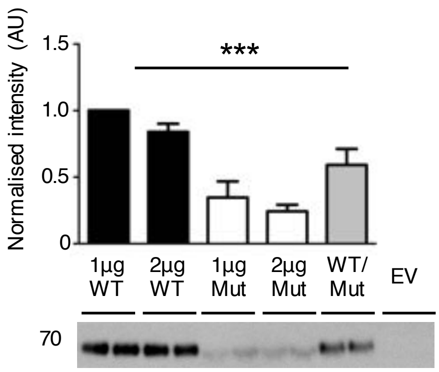

Fig 5d. Secretion of low molecular weight adiponectin complexes into the culture medium of HEK293-T cells transfected with 1µg or 2µg wild type (black) or mutant (white) *ADIPOQ*, or co-transfected with 1µg wild type and 1µg mutant *ADIPOQ* (grey), determined by non-denaturing, non-reducing SDS-PAGE followed by western blotting of adiponectin.

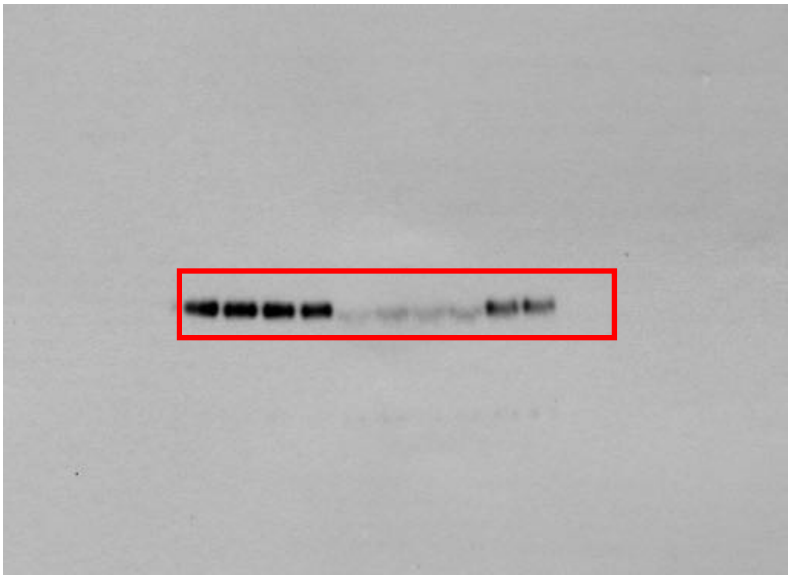

Ad 009

Full unedited gels for Supplementary Figure 2a

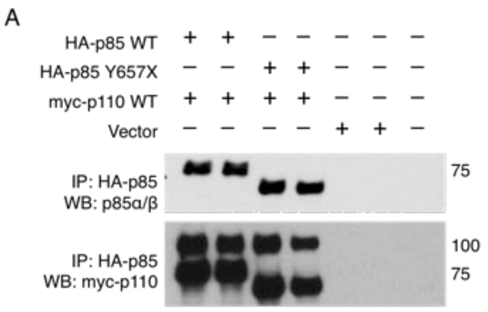

Supp Fig 2a. Anti-HA immunoprecipitates of HEK293-T cells transiently overexpressing HA-tagged WT or mutant p85α and myc-tagged WT p110α subject to western blotting first against HA (upper panel) and subsequently against myc (lower panel, upper bands; lower bands represent residual signal from the anti-HA antibodies).

1 2 3 4 5 6 7

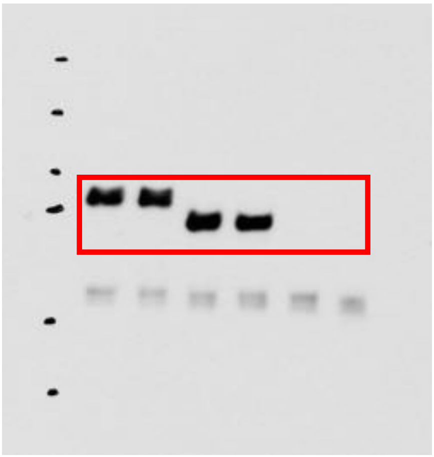

IP: HA

WB: Total p85

1 2 3 4 5 6 7

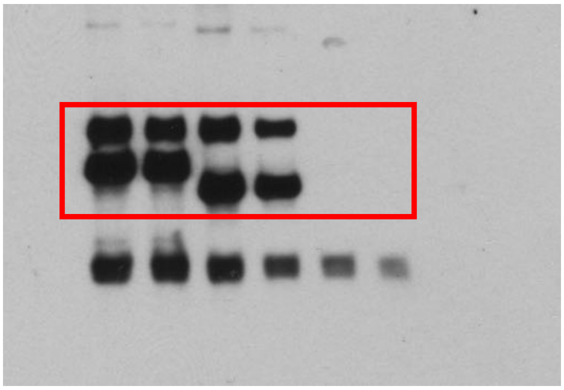

IP: HA

WB: myc

1) **WT p85 + WT p110, -ins**
2) **WT p85 + WT p110, +ins**
3) **Mutant p85 Y657X + WT p110, -ins**
4) **Mutant p85 Y657X + WT p110, +ins**
5) **Empty vector, -ins**
6) **Empty vector, +ins**
7) **Lysate + beads only**

ID: PI3K_041

Full unedited gels for Supplementary Figure 2b

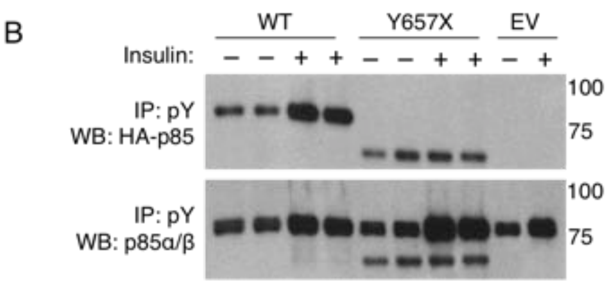

Supp Fig 2b. Western blot of HA-tagged p85α (upper panel) and total p85 (endogenous and recombinant; lower panel) in phosphotyrosine immunoprecipitates of lysates from HEK293-T cells transiently overexpressing HA-tagged wild type or mutant p85a and myc-tagged p110a (or empty vector), treated with PBS or 100nM insulin for 5 minutes. Representative blots shown from three independent experiments.

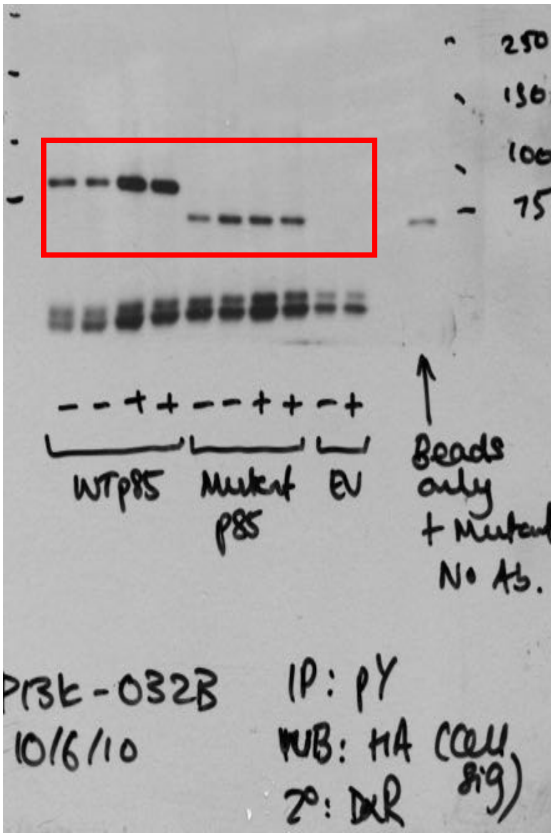

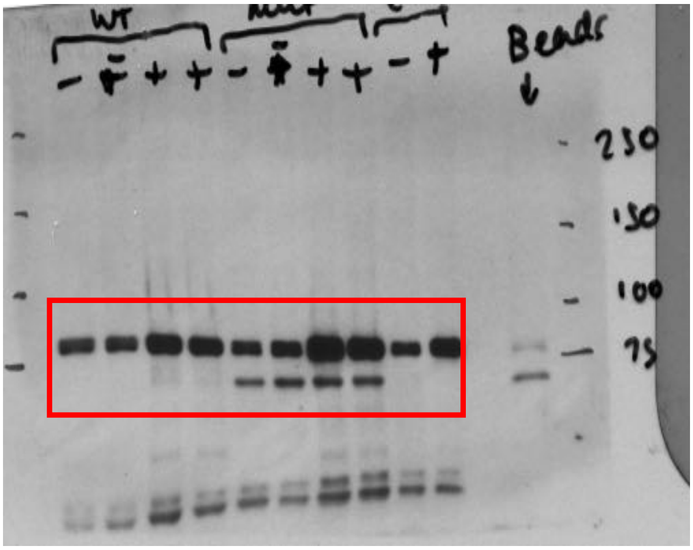

IP: pY
WB: HA

IP: pY
WB: Total p85

ID: PI3K_032

Full unedited gels for Supplemental Figure 3

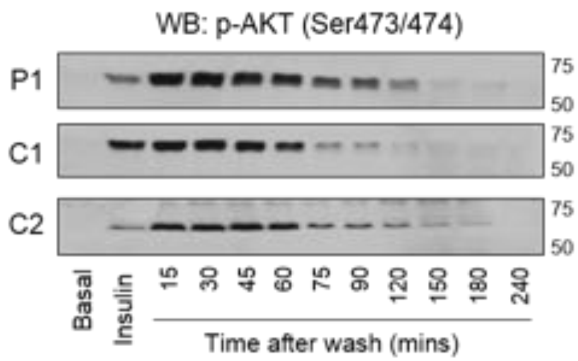

Supp Fig 3. AKT dephosphorylation in primary dermal fibroblasts: Western blot of phosphorylated AKT at serine 473/474 in dermal fibroblasts from P1 and two healthy controls (C1, 2) serum-starved overnight, stimulated with 10nM insulin for 5 minutes, washed twice in warm PBS then incubated at 37°C for the times indicated. Representative example of two independent experiments.

P1
pAKT

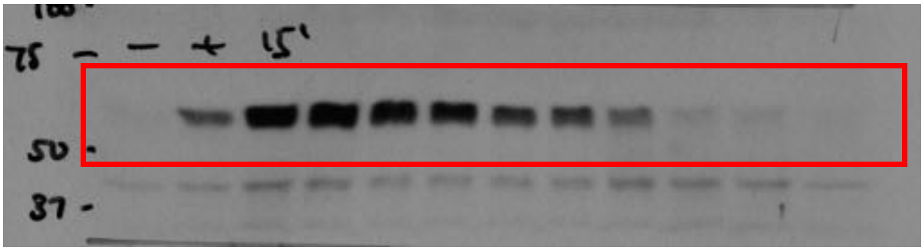

C1
pAKT

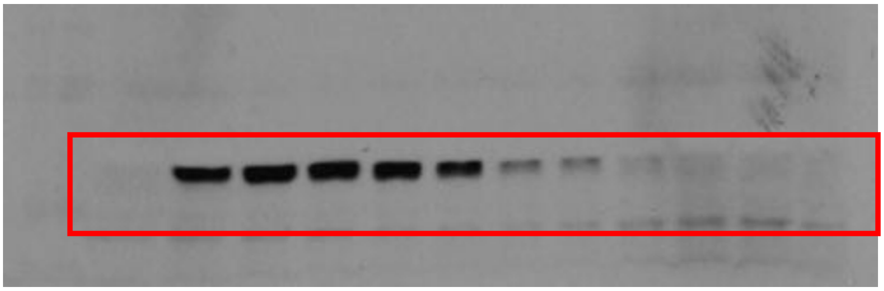

C2
pAKT

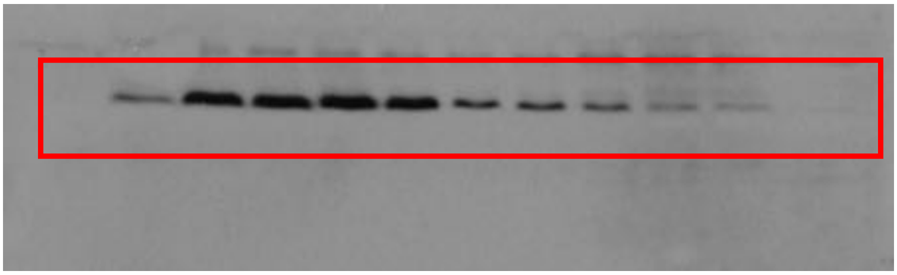

Full unedited gels for Supplemental Figure 4

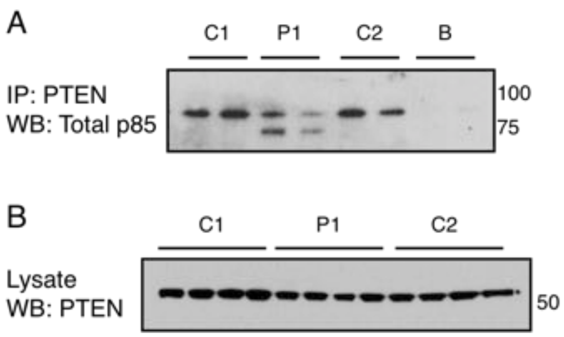

Supp Fig 4. PTEN expression and association with p85 in primary dermal fibroblasts: (A) Western blot of total p85 in anti-PTEN immunoprecipitates from dermal fibroblasts of P1 and two healthy controls (C1, C2). Lane marked "*B*" indicates beads only control, without PTEN antibody. (B) Western blot of PTEN in fibrobrast lysates from P1 and two healthy controls (C1, C2).

IP: PTEN
WB: Total p85

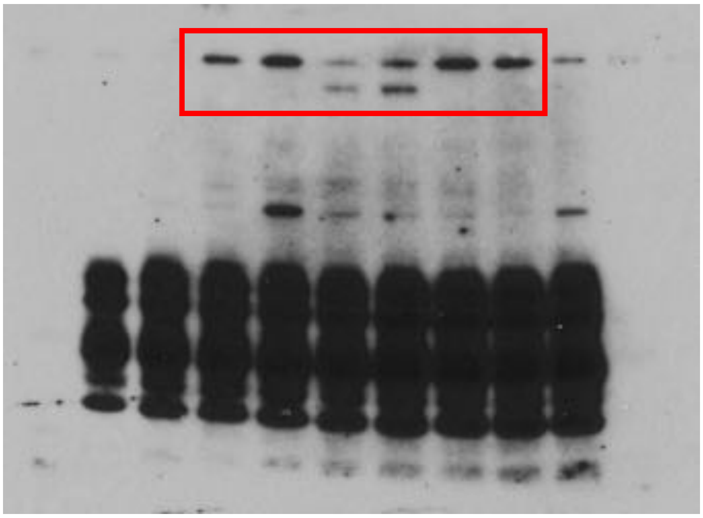

Lysate
WB: PTEN

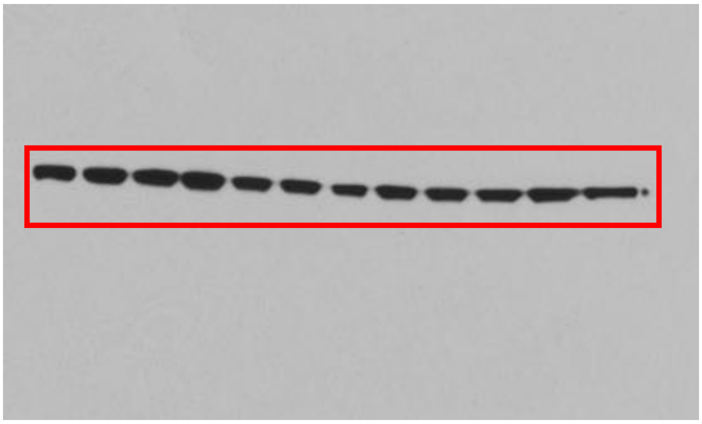

Supplement: Supplemental data [file jciinsight-1-88766-s001.pdf]
